# Takotsubo syndrome outcomes predicted by thyroid hormone signature: insights from cluster analysis of a multicentre registry

**DOI:** 10.1016/j.ebiom.2024.105063

**Published:** 2024-03-18

**Authors:** Assem Aweimer, Johannes W. Dietrich, Francesco Santoro, Mireia Camins Fàbregas, Andreas Mügge, Iván J. Núñez-Gil, Ravi Vazirani, Oscar Vedia, Toni Pätz, Ilaria Ragnatela, Luca Arcari, Massimo Volpe, Miguel Corbì-Pascual, Manuel Martinez-Selles, Manuel Almendro-Delia, Alessandro Sionis, Aitor Uribarri, Holger Thiele, Natale Daniele Brunetti, Ingo Eitel, Thomas Stiermaier, Nazha Hamdani, Mohammad Abumayyaleh, Ibrahim Akin, Ibrahim El-Battrawy

**Affiliations:** a Cardiology and Angiology Department, Medical Hospital II, Bergmannsheil University Hospitals, Ruhr University of Bochum, Bochum, NRW, Germany; b Diabetes, Endocrinology and Metabolism Section, Department of Medicine I, St. Josef University Hospital, Ruhr University of Bochum, Bochum, NRW, Germany; c Diabetes Centre Bochum/Hattingen, St. Elisabeth Hospital Blankenstein, Im Vogelsang 5-11, Hattingen 45527, Germany; d Centre for Rare Endocrine Diseases, Ruhr Centre for Rare Diseases (CeSER), Ruhr University Bochum and Witten/Herdecke University, Alexandrinenstr. 5, Bochum 44791, Germany; e Centre for Diabetes Technology, Catholic Hospitals Bochum, Gudrunstr. 56, Bochum 44791, Germany; f University of Foggia, Department of Medical and Surgical Sciences, Foggia, Italy; g Hospital Clínico Universitario San Carlos, Interventional Cardiology, Cardiovascular Institute, Madrid, Spain; h University Heart Center Lübeck, Medical Clinic II (Cardiology/Angiology/Intensive Care Medicine) and German Center for Cardiovascular Research (DZHK), Partner Site Hamburg/Kiel/Lübeck, Lübeck, Germany; i Institute of Cardiology, Madre Giuseppina Vannini Hospital, Rome, Italy; j Department of Clinical and Molecular Medicine, University of Rome Sapienza and IRCCS San Raffaele Rome, Italy; k Department of Cardiology, Complejo Hospitalario de Albacete, Albacete, Spain; l Department of Cardiology, Hospital General Universitario Gregorio Marañon, CIBERCV, Madrid, Spain; m Servicio de Cardiología, Hospital Virgen de la Macarena, Sevilla, Spain; n Cardiology Service, Vall d’Hebron, University Hospital, Barcelona, Spain; o Heart Center Leipzig at University of Leipzig and Leipzig Heart Science, Department of Internal Medicine/Cardiology, Leipzig, Germany; p Institut für Forschung und Lehre (IFL), Department of Molecular and Experimental Cardiology, Ruhr-University Bochum, Bochum, Germany; q Institute of Physiology, Ruhr-University Bochum, Bochum, Germany; r First Department of Medicine, Faculty of Medicine University Medical Centre Mannheim (UMM), University of Heidelberg, Mannheim, Germany; s Centre for Thyroid Medicine, Catholic Hospitals Bochum, Gudrunstr. 56, Bochum 44791, Germany; t CIBERCV, Madrid, Spain; u Universidad Europea, Universidad Complutense, Madrid, Spain

**Keywords:** Takotsubo syndrome, Thyroid, Stress cardiomyopathy, Thyrotoxicosis

## Abstract

**Background:**

Recently, abnormal thyroid function was shown to be common in patients with Takotsubo syndrome (TTS), being classified into “endocrine-type” and “stress-type” responses. The aim of this study was to investigate the association between thyroid homeostasis and TTS in a larger international registry.

**Methods:**

In total 288 patients with TTS were enrolled through the GEIST multicentre registry from Germany, Italy and Spain. Thyrotropin (TSH), free T4 (FT4) and free T3 (FT3) concentrations were analysed at admission. Data were collected both retrospectively and prospectively from 2017 onwards. Primary endpoints included in-hospital and all-cause fatality, determined by cluster analysis using an unsupervised machine learning algorithm (k-medoids).

**Findings:**

Three clusters were identified, classifying TTS with low (TSLT), high (TSHT) and normal (TSNT) thyroid output, based on TSH and FT4 levels in relation to the median thyroid’s secretory capacity (SPINA-GT). Although TSH and FT4 concentrations were similar among survivors and non-survivors, these clusters were significantly associated with patient outcomes. In the longitudinal Kaplan–Meier analysis including in- and out-of-hospital survival, the prognosis related to concentrations of TSH, FT4, and FT3 as well as SPINA-GT, deiodinase activity (SPINA-GD) and clusters. Patients in the TSHT cluster and with cardiogenic shock had a lower initial left ventricular ejection fraction (LVEF).

**Interpretation:**

This study suggests that thyroid hormones may impact the evolution and prognosis of TTS. The findings indicate that thyroid-derived biomarkers may help identify high-risk patients and pave the way for novel personalized and preventive therapeutic options.

**Funding:**

This research was not funded by any public, commercial, or not-for-profit agencies.

**Research in context**

**Evidence before this study**

Prior evidence suggests that hyper- and hypothyroidism may act as triggers for Takotsubo syndrome (TTS), with thyrotoxicosis being the most common thyroid alteration associated with TTS. A previous study demonstrated myocardial stunning in a patient with TTS and thyrotoxicosis. However, hypothyroidism also appears to have an increased odds ratio in TTS. A recent small observational study found a high proportion of abnormal thyroid function in patients with TTS and proposed categorizing them into “endocrine-type” (hyperthyroidism, thyrotoxicosis) and “stress-type” (elevated set point for free T4 concentration in type 2 allostatic load) responses. The current study aims to investigate the relationship between thyroid homeostasis and TTS in a large multicentre international registry, specifically focusing on thyroid hormone profiles at admission and their association with outcome parameters and fatality of TTS.

**Added value of this study**

In our analysis, we included 288 patients diagnosed with TTS, all with comprehensive thyroid profiles, from the international TTS registry (GEIST). Three clusters were identified in this study, categorizing patients with TTS based on their thyroid output: TTS with low thyroid output (TSLT), TTS with high thyroid output (TSHT), and TTS with normal thyroid output (TSNT). Cluster TSLT was associated with significant better survival than clusters TSHT and TSNT. Although TSH and FT4 concentrations were similar between survivors and non-survivors, these clusters had a significant impact on predicting fatality. Non-survivors had significantly lower FT3 concentration. In a longitudinal analysis using the Kaplan–Meier method, the concentrations of TSH, FT4, FT3, SPINA-GT, SPINA-GD (deiodinase activity), and the identified clusters all played a role in predicting prognosis, including in-hospital and out-of-hospital survival. Multivariable analysis showed that FT3 and LVEF were predictors of cardiogenic shock.

**Implications of all the available evidence**

The present study demonstrates that thyroid hormones play a significant role in the evolution of TTS and its prognosis. It might, therefore, pave the way to novel therapeutic options. Innovative thyroid-derived biomarkers may help to identify high-risk patients with TTS and promote a personalized and preventive use of several drugs to reduce all-cause fatality.

## Introduction

Stress cardiomyopathy, or Takotsubo syndrome (TTS), was first described in 1990 when patients were admitted with symptoms, ECG changes and altered laboratory parameters mimicking patients with myocardial infarction. TTS has four types (apical, midventricular, basal, and focal) with varied outcomes, lacks specific coronary angiography findings, and has a 4% recurrence rate.^1^^,^^2^ Complications include atrial fibrillation, other form of arrhythmia, cardiogenic shock, stroke and thromboembolic events, and respiratory failure.^3^, ^4^, ^5^, ^6^, ^7^ Two-thirds of TTS cases are triggered by severe emotional or physical stress, and hormonal changes in post-menopausal women may contribute to pathogenesis.^1^^,^^8^, ^9^, ^10^, ^11^, ^12^, ^13^ Adrenergic system activation is a key mediator, supported by elevated catecholamine levels. Rat models suggest high epinephrine levels trigger cardio-depression, potentially serving as a cardioprotective strategy. Altered beta-adrenoceptors in human myocardial tissue may protect against catecholamine toxicity in TTS.^14^, ^15^, ^16^, ^17^, ^18^

Several case reports have suggested other triggers of TTS, including hyper- and hypothyroidism.^12^^,^^19^ The most frequent thyroid alteration linked to TTS is thyrotoxicosis due to various disease entities.^20^, ^21^, ^22^, ^23^ Previously Miyazaki et al. visualized myocardial stunning by scintigraphy in a patient with TTS and concomitant thyrotoxicosis.^24^ However, in TTS the odds ratio of hypothyroidism seems to be increased, too.^25^

The exact pathogenesis of this relationship is not yet fully understood. It was observed that thyroid hormones sensitize the myocardial tissue to the effects of catecholamines^24^^,^^26^^,^^27^ which was suggested to be one of the reasons for increased cardiovascular mortality in thyrotoxicosis.^28^ This mechanism may be an important mechanism for the evolution of TTS as well.^24^ It would explain an increased risk of TTS in stress situations in the presence of elevated thyroid hormone concentrations.

Recently, we observed in a small multicentre observational study, with 16 patients with TTS included in the analysis, a high proportion of abnormal thyroid function.^29^ We suggested a categorization of patients with TTS in “endocrine-type” response (hyperthyroidism, thyrotoxicosis) and “stress-type” response (elevated set point for free T4 concentration in type 2 allostatic load).

The aim of the present study was to investigate the association between thyroid homeostasis and TTS in a large multicentre international registry comprising patients with TTS from the German Italian and Spanish Takotsubo (GEIST)-Registry at the TTS event. In addition, we analysed thyroid hormone profiles at the time of admission with respect to outcome parameters and defined homeostatic signatures, which are associated with the fatality of TTS.

## Methods

In total 690 patients with TTS were screened for eligibility from centers participating in the GEIST multicentre registry, including subjects from Germany, Italy and Spain (clinical trial. gov = NCT04361994). Sex was collected as self-reported by study participants. Briefly, data were collected partially retrospectively and partially prospectively from 2017 onwards. Inclusion criteria for cluster analysis was the availability of TSH and FT4 concentration and for survival analysis the availability of outcome data.

TTS was diagnosed using the current diagnostic criteria from the European position statement^30^: I) transient regional wall motion abnormalities which extend beyond a single epicardial vascular distribution, II) the absence of culprit atherosclerotic coronary artery disease including acute plaque rupture, thrombus formation, coronary dissection or other changes related to transient ventricular dysfunction, III) new and transient electrocardiographic changes, IV) positive but relatively small elevation in cardiac enzyme markers including troponin I and NT-pro-BNP and V) recovery of ventricular systolic function at follow-up. If the diagnosis of TTS was uncertain and other differential diagnoses, e.g., myocarditis or other forms of structural heart diseases, were possible a cardiac magnetic resonance tomography was performed to confirm the diagnosis and to exclude other cardiac diseases.

The minimal standard acquisition protocol for transthoracic echocardiography was required in included patients.^31^ After the TTS event, a follow-up echocardiography was performed before discharge and at follow-up presenting a normalization of LV function and absence of typical TTS pattern.

The pattern of thyroid function was classified as follows: Normal thyroid function was defined as normal TSH, FT4 and FT3 concentrations; TACITUS (**t**hyroid **a**llostasis in **c**ritical **i**llness, **t**umours, **u**raemia and **s**tarvation, i.e., type 1 thyroid allostatic load): reduced FT3 concentration; type 2 thyroid allostatic load: increased TSH and FT4 concentrations; primary thyrotoxicosis: reduced TSH and increased FT4 concentrations. In order to get exploratory information on pathophysiological processes, derived parameters of thyroid homeostasis were calculated, including thyroid’s secretory capacity (SPINA-GT), total deiodinase activity (SPINA-GD), thyrotroph thyroid hormone resistance index (TTSI) and Jostel’s TSH index (JTI), as previously described.^32^, ^33^, ^34^ As an additional measure for the set point of thyroid hormones, the thyroid feedback quantile-based index (TFQI) was calculated, as recently suggested.^35^ SPINA-GT was only calculated in subjects not on levothyroxine substitution therapy. The equations used are provided along with required parameters in the Supplementary appendix.

The primary outcome in our study was in- and out-of-hospital, all-cause fatality. The secondary outcome was a composite end-point of in-hospital complications (arrhythmias, thromboembolic events or stroke, pulmonary congestion, cardiogenic shock with use of catecholamine and/or assist device) and was assessed at the time of the TTS index-event. In addition, the recurrence of TTS after the initial event was documented. The TTS cohort was followed up by outpatient evaluation and/or systematic telephone interview.

### Statistics

An unsupervised machine learning (ML) algorithm (partitioning about medoids or PAM based on k-medoids) was used to find patterns in the affine space of TSH and FT4 concentrations. Medoids are defined as representative data points in a cluster whose sum of dissimilarities to all data points in the respective cluster is minimal.^36^ The number of medoids to be generated (k) was selected based on silhouette scores for Euclidian distances of the data points to the respective cluster medoid. Selected was the k value with the highest average silhouette width $\bar{s}$. The plausibility of the results was visually checked with dendrograms.

Sample size calculations were performed with Monte Carlo simulations of the empirical power of a Wilcoxon–Mann–Whitney test (see Supplementary appendix for details). Data are presented as median with interquartile range (IQR) for continuous variables with a non-normal distribution, and as frequency (%) for categorical variables. Mean ± standard deviation (SD) or median (1st–3rd quartile) are presented for continuous variables, depending on the type and symmetry of distribution. Ratios of medians are reported along with 95% confidence intervals. Normal distribution was assessed using the Kolmogorov–Smirnov test and Q–Q plots, the homogeneity of variances with an F-test. Since the conditions for a t-test were not met the U-test according to Wilcoxon, Mann and Whitney was used to compare continuous variables between groups. Categorical variables were compared using the Chi-squared-test or, if the expected value in at least one cell of the contingency table was below 5, Fisher’s exact test. Phi statistics and Youden’s J from receiver operating characteristic (ROC) curves were used to obtain optimal cut-off values of measured and calculated parameters of thyroid homeostasis with respect to survival. A log-rank test was used to compare the Kaplan–Meier survival curves of patients with TTS, differentiating those with thyroid parameters below and above the predetermined cutoff value. The day of onset of TTS was specified as the origin and start time of the survival analysis. The date of death or right censoring during follow-up was defined as the end time. Univariate Cox regression analysis was performed for a set of predefined candidate predictors (TSH, FT4, FT3, age, sex, hypertension, diabetes mellitus, hypercholesterolemia, smoking, atrial fibrillation, malignancy, neurological disease, psychiatric disease, stressful, physical and emotional triggers, creatinine, apical, midventricular and basal ballooning, focal ballooning, and initial EF). For each potential predictor, proportionality was tested based on scaled Schoenfeld residuals. For continuous predictors, linearity was assessed based on plots of Martingale residuals against the respective covariate. Factors with p < 0.10 on univariate analysis were entered into a multivariable Cox regression analysis to identify independent risk factors for prognosis. An initial maximal model was simplified in a step-wise manner by repeatedly eliminating the least significant predictor to deliver a minimal model containing significant (p < 0.5) predictors only. In order to avoid spurious correlations, calculated parameters of thyroid homeostasis and medication were not included in multivariable analyses. An additional hierarchical Cox regression model was derived based on sex and age as potentially confounding frailty clusters. Statistical analysis was performed using R for macOS version 4.2.3 with the packages ROCR, SPINA, survival, coxme, cluster and factoextra, and SPSS 23.0. A two-tailed p < 0.05 was considered to be statistically significant. All p values ≥ 0.0001 are reported with two significant digits.

### Ethics

The study was conducted according to Good Clinical Practice and the Declaration of Helsinki. All patients provided written informed consent before inclusion in the registry, which meets the requirements of the respective local ethics committees (Ruhr University Bochum (Germany), registration number: 22-7684; University of Lübeck (Germany); registration number: 19-341; University of Mannheim (Germany); registration number: 2015-841R-MA; University of Foggia (Italy), registration number 19/CE/2019; University of Madrid (Spain), registration number 11/349-E).

### Role of funders

This research was not funded by any public, commercial, or not-for-profit agencies.

## Results

### Study population

**Fig. 1:**
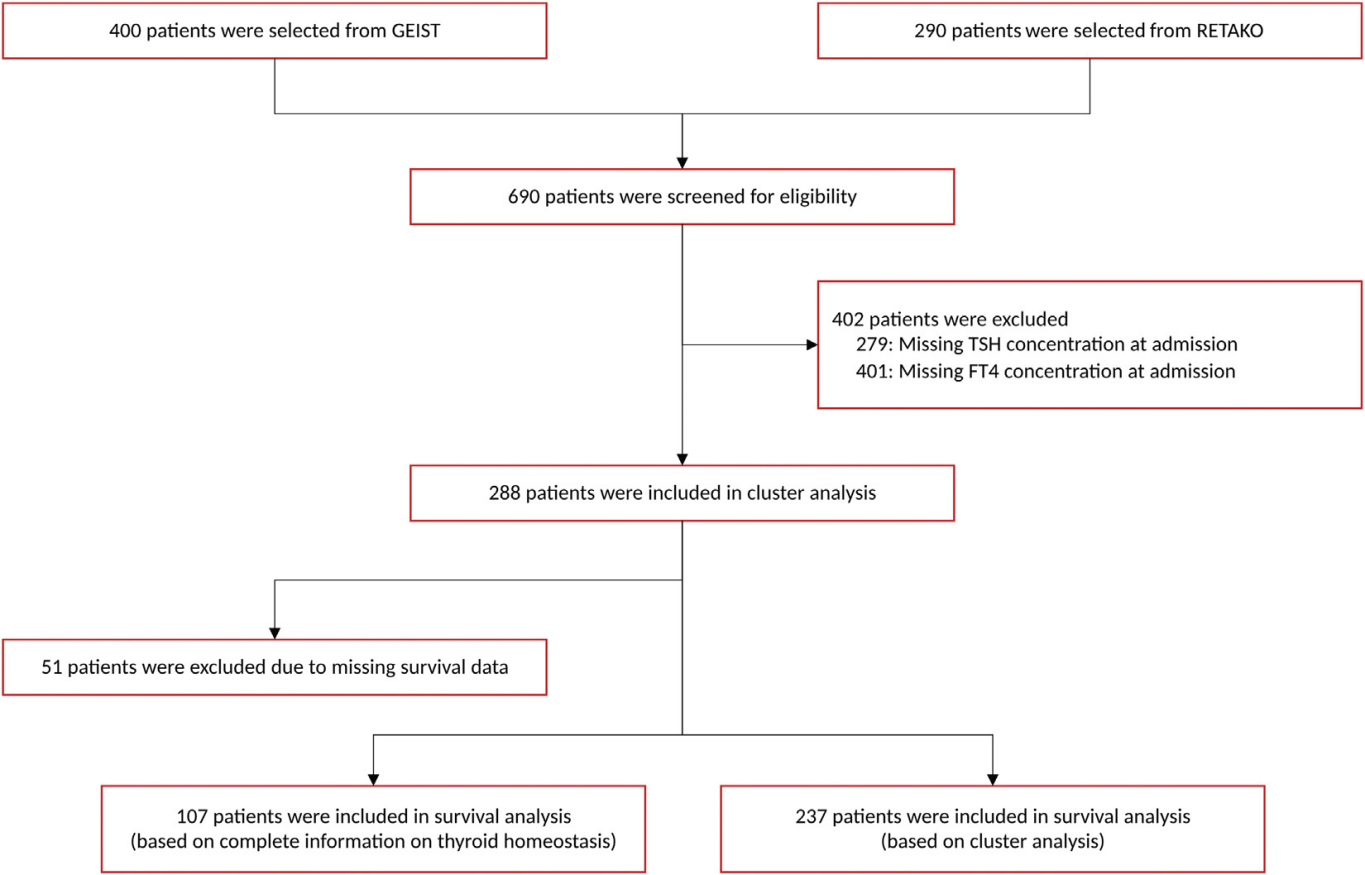
Flow chart of screened and included cases.

From 690 datasets available in the GEIST registry 288 cases with a complete thyroid panel could be included in the cluster analysis; 237 persons could be followed up based on clusters and survival data (Fig. 1). The median long-term follow-up was 4.3 years (IQR 486–2854 days).

Only 25.2% of the included persons had normal thyroid function, 40.5% were classified as TACITUS-syndrome, 16.0% had a pattern of type 2 allostatic load, 2.3% suffered from hypothyroidism and 16.0% presented with primary thyrotoxicosis.

24 persons were faced with recurrent TTS, resulting in a low recurrence rate of 4.7%. None of the investigated variables predicted recurrence.

### Primary endpoint: survival

#### Survivors vs. non-survivors

Among the cohort of subjects receiving a full thyroid panel 41 (31.3%) patients died over follow-up of up to 6597 days. Non-survivors tended to be older (71.1 ± 14.4 vs. 68.2 ± 11.6 years; p = 0.06 [Wilcoxon–Mann–Whitney test]), and suffered more frequently from malignancy (22.0% vs. 5.5%; p = 0.01 [Chi-squared-test]). The rate of cardiovascular risk factors e.g., arterial hypertension, Diabetes mellitus, smoking and hypercholesterolemia were similar in survivors and non-survivors. Likewise, comorbidities including psychiatric and neurological diseases as well as coronary artery disease were not different between survivors and non-survivors. Non-survivors expressed a lower left ventricular ejection fraction (38.4 ± 15.2% vs. 45.2 ± 12.5%; p = 0.003 [Wilcoxon–Mann–Whitney test]). Additional information on the characteristics of the study population based on thyroid function testing is provided in Table 1. Information on the total population of the GEIST registry (including subjects that did not receive functional thyroid testing) is reported in the Supplementary Table S3.

**Table 1:** Basic characteristics of the study population that received a full panel of thyroid investigation.

	Survivors (n = 90)	Non-survivors (all-cause fatality; n = 41)	p
Age (years)	68.2 ± 11.6	71.1 ± 14.4	0.064
Female (%)	82 (91.1%)	33 (80.5%)	0.15
Male (%)	8 (8.9%)	(8) (19.5%)	
BMI (kg/m^2^)	24.9 ± 6.9	21.7 ± 3.2	0.11
Hypertension (%)	48 (53.3%)	27 (65.9%)	0.25
Diabetes mellitus (%)	10 (11.1%)	7 (17.1%)	0.51
Atrial fibrillation (%)	16 (17.8)	9 (22.0)	0.75
Hypercholesterolemia (%)	22 (24.4%)	10 (24.4%)	1.00
Smoking (%)	20 (22.2%)	12 (29.3%)	0.52
Coronary artery disease (%)	11 (12.2%)	11 (26.8%)	0.068
Malignancy (%)	5 (5.5%)	9 (22.0%)	0.011
Neurological disease (%)	24 (26.7%)	8 (19.5%)	0.51
Psychiatric disease (%)	10 (11.1%)	4 (9.8%)	1.00
Stressful trigger (%)	66 (73.3%)	28 (68.3%)	0.70
Physical trigger (%)	27 (30.0%)	19 (46.3%)	0.11
Emotional trigger (%)	39 (43.3%)	10 (24,4%)	0.060
Creatinine concentration (μmol/L)	79.6 (70.7–92.4)	101.7 (73.2–120.2)	0.039
Apical ballooning (%)	61 (67.8%)	35 (85.4%)	0.058
Midventricular ballooning (%)	28 (31.1%)	4 (9.8%)	0.016
Basal ballooning (%)	1 (1.1%)	2 (4.9%)	0.23
Initial EF (%)	45.2 ± 12.5	38.4 ± 15.2	0.0036
Antihypertensive drugs (%)	75 (83.3%)	31 (75.6%)	0.42
ACE-I or ARB (%)	54 (60.0%)	21 (51.2%)	0.45
Beta blockers (%)	62 (68.9%)	21 (51.2%)	0.080
Diuretics (%)	29 (32.2%)	16 (39.0%)	0.57
Aldosterone antagonists (%)	5 (5.7%)	2 (4.9%)	1.00
Statins (%)	37 (41.1%)	15 (36.6%)	0.77
Amiodarone (%)	5 (5.6%)	0 (0%)	0.32
Anticoagulation (%)	33 (37.5%)	14 (34.1%)	0.86
Antidiabetic medication (%)	8 (9.2%)	3 (8.1%)	1.00
Thyroid medication (%)	23 (25.6%)	9 (21.9%)	0.82
Levothyroxine (%)	14 (15.6%)	2 (4.9%)	0.15
Perchlorate (%)	3 (3.3%)	3 (7.3%)	0.38
Iodine (%)	1 (1.1%)	0 (0%)	1.00
Antithyroid agents (%)	1 (1.1%)	0 (0%)	1.00

Data are reported as mean ± SD, median (1st–3rd quartile) or count (percentage).

BMI, body mass index; EF, left ventricular ejection fraction.

The comparison of subjects with and without measurement of thyroid hormones revealed no difference in survival rate and important structural data including age, sex, hypertension, diabetes, EF, ballooning and kind of trigger. Not unexpectedly, thyroid medication was more common in persons who received a laboratory assessment of thyroid function (Supplementary Table S2). The basic characteristics and the distribution of thyroid hormones were similar in the subpopulation that received a full panel of thyroid investigation (i.e., measurement of TSH, FT4, **and** FT3) to those of the total population (Table 1 and Supplementary Tables S3 and S4). However, the statistical power is lower in the subgroup, which leads to a slightly lower number of significant results.

#### Cluster analysis

**Fig. 2:**
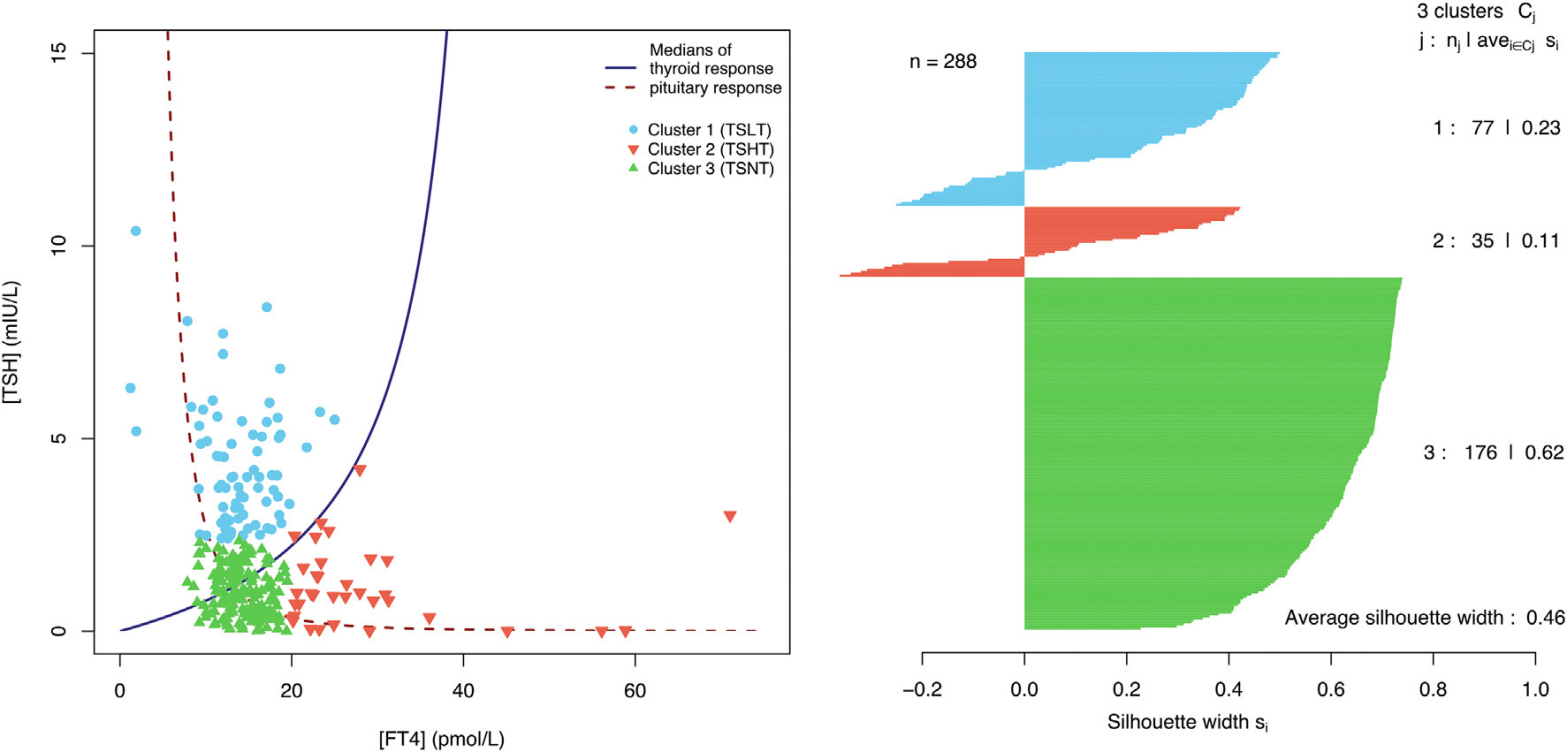
Definition of clusters. Thyroid response and pituitary response curves denote the median of thyroid’s secretory capacity (SPINA-GT) and empirical thyrotropic pituitary response.

Based on the TTS cohort, the PAM algorithm identified three clusters in the affine space of TSH and FT4 concentrations (Fig. 2). Cluster 1, including 77 persons (27%), was characterized by moderately high TSH and low to normal FT4 concentrations and was labelled as TTS with low thyroid output (TSLT). Cluster 2, labelled as TTS with high thyroid output (TSHT) and including 35 patients (12%), was characterized by low to slightly elevated and normal TSH concentrations and slightly to moderately elevated FT4 concentration. Cluster 3 (labelled as TTS with normal thyroid output or TSNT) included 176 subjects (61%) and was marked by normal TSH and FT4 concentrations. Data points of the TSHT cluster were located on or above the population-derived 50% percentile of SPINA-GT, the points of the TSLT cluster were without exception below the median of SPINA-GT.

In contrast to marked differences in thyroid function, the clusters were similar and homogeneous (Table 2). However, subjects in TSHT cluster were older and expressed lower EF than members of the other two clusters. In addition, physical triggers were more frequently described in cluster 2.

**Table 2:** Characteristics of the three clusters. The figures in the first two lines refer to medoids of TSH and FT4 concentration as identified by the clustering algorithm.

	Cluster 1 (TSLT; n = 77)	Cluster 2 (TSHT; n = 35)	p compared to cluster 1	Cluster 3 (TSNT; n = 176)	p compared to cluster 1	p compared to cluster 2
TSH medoid (mIU/L)	3.74	0.91		1.02		
FT4 medoid (pmol/L)	13.8	24.8		14.2		
TSH (mIU/L)	2.8 (2.9–5.1)	0.9 (0.4–1.7)	<0.0001	1.0 (0.4–1.5)	<0.0001	0.95
FT4 (pmol/L)	13.5 (11.8–17.1)	24.3 (22.2–28.6)	<0.0001	14.1 (12.5–16.1)	0.59	<0.0001
FT3 (pmol/L)	3.9 (2.9–4.5)	4.5 (2.5–5.8)	0.030	4.0 (2.7–4.6)	0.79	0.030
SPINA-GT (pmol/s)	1.9 (1.5–2.1)	7.6 (5.1–11.3)	<0.0001	4.2 (2.8–7.8)	<0.0001	<0.0001
SPINA-GD (nmol/s)	22.4 (16.7–29.8)	16.2 (12.7–21.2)	0.0012	25.6 (17.6–30.5)	0.96	0.0012
JTI	3.1 (2.8–3.6)	3.4 (2.4–3.9)	0.55	1.7 (1.2–2.2)	<0.0001	<0.0001
TTSI	171.4 (132.1–217.3)	77.7 (24.9–127.8)	<0.0001	41.2 (21.8–66.6)	<0.0001	0.0020
TFQI	0.8 (0.6–0.9)	0.3 (0.0–0.5)	<0.0001	0.1 (0.0–0.2)	<0.0001	0.029
Age (years)	71.1 ± 11.1	76.7 ± 9.9	0.026	69.3 ± 13.3	0.49	0.0080
Female (%)	70 (91%)	31 (89%)	1.00	155 (88%)	1.00	1.00
Male (%)	7 (9%)	4 (11%)		21 (12%)		
BMI (kg/m^2^)	24.1 ± 4.5	27.0 ± 6.4	0.80	23.5 ± 6.3	0.80	0.49
Hypertension (%)	49 (64%)	23 (66%)	1.00	114 (65%)	1.00	1.00
Diabetes mellitus (%)	12 (16%)	10 (29%)	0.38	35 (20%)	0.48	0.39
Atrial fibrillation (%)	13 (17%)	11 (31%)	0.13	26 (15%)	0.71	0.081
Hypercholesterolemia (%)	30 (39%)	16 (46%)	0.54	59 (33%)	0.54	0.53
Smoking (%)	12 (16%)	2 (6%)	0.33	35 (20%)	0.48	0.15
Coronary artery disease (%)	12 (16%)	3 (9%)	0.70	24 (14%)	0.70	0.70
Malignancy (%)	11 (14%)	5 (14%)	1.00	12 (7%)	0.25	0.25
Neurological disease (%)	15 (19%)	4 (11%)	0.42	44 (25%)	0.42	0.36
Psychiatric disease (%)	4 (5%)	2 (6%)	1.00	20 (11%)	0.49	0.81
Stressful trigger (%)	50 (65%)	28 (80%)	0.38	125 (71%)	0.38	0.38
Physical trigger (%)	24 (31%)	20 (57%)	0.018	54 (31%)	1.00	0.011
Emotional trigger (%)	25 (32%)	8 (32%)	0.37	74 (42%)	0.25	0.11
Creatinine concentration (μmol/L)	89.7 (74.3–124.0)	91.9 (72.7–106.6)	0.71	81.3 (69.8–98.6)	0.11	0.71
Apical ballooning (%)	56 (73%)	32 (89%)	1.00	130 (74%)	1.00	1.00
Midventricular ballooning (%)	16 (21%)	2 (6%)	0.080	36 (20%)	0.080	1.00
Basal ballooning (%)	3 (0%)	0 (0%)	0.83	1 (0%)	1.00	0.26
Initial EF (%)	45.2 ± 12.5	38.4 ± 11.3	0.021	44.5 ± 13.6	0.87	0.019

TSH, Thyroxine stimulating hormone; FT4, free thyroxine; FT3, free triiodothyronine SPINA-GT, thyroid’s secretory capacity, SPINA-GD, sum activity of peripheral deiodinases; JTI, Jostel’s TSH index; TTSI, thyrotroph thyroid hormone resistance index (TTSI); TFQI, thyroid feedback quantile-based index (TFQI); BMI, body mass index; EF, left ventricular ejection fraction. Data are reported as mean ± SD, median (1st–3rd quartile) or count (percentage).

#### Markers of thyroid function and classes of thyroid function among non-survivors and survivors

TSH and FT4 concentrations were similar between survivors and non-survivors (Table 3). Non-survivors showed significantly lower FT3 concentration (3.3; 1.5–3.9 pmol/L vs. 4.1; 3.2–4.6 pmol/L; p = 0.01 [Wilcoxon–Mann–Whitney test]; ratio of medians: 0.81; 0.59–0.96) compared to survivors. Whereas SPINA-GT was significantly higher among non-survivors compared to survivors (3.8; 2.7–7.1 pmol/s vs. 2.9; 2.0–5.8 pmol/s; p = 0.02 [Wilcoxon–Mann–Whitney test]; ratio of medians: 1.34; 1.03–1.64), SPINA-GD was significantly lower in non-survivors (18.9 ± 10.0 nmol/s vs. 24.1 ± 9.8 nmol/s; p = 0.005 [Wilcoxon–Mann–Whitney test]; ratio of medians: 0.72; 0.56–0.89). Markers of central control (JTI, TTSI, and TFQI) were similar between survivors and non-survivors.

**Table 3:** Markers of thyroid function in the study population.

	Survivors	Non-survivors (all-cause fatality)	p
TSH (mIU/L), n = 411	1.4 (0.7–2.6)	1.3 (0.5–1.8)	0.051
FT4 (pmol/L), n = 289	14.0 (12.1–16.5)	14.5 (12.8–17.1)	0.11
FT3 (pmol/L), n = 169	4.1 (3.2–4.6)	3.3 (1.5–3.9)	0.012
SPINA-GT (pmol/s), n = 269	2.9 (2.0–5.8)	3.8 (2.7–7.1)	0.015
SPINA-GD (nmol/s), n = 169	26.3 (16.5–30.5)	18.9 (11.3–25.0)	0.0053
JTI, n = 288	2.2 (1.5–2.9)	2.0 (1.3–2.6)	0.090
TTSI, n = 288	62.9 (28.3–119.5)	51.4 (22.5–85.7)	0.069
TFQI, n = 288	0.27 (0.03–0.63)	0.25 (0.02–0.49)	0.14

TSH, Thyroxine stimulating hormone; FT4, free thyroxine; FT3, free triiodothyronine SPINA-GT, thyroid’s secretory capacity, SPINA-GD, sum activity of peripheral deiodinases; JTI, Jostel’s TSH index; TTSI, thyrotroph thyroid hormone resistance index (TTSI); TFQI, thyroid feedback quantile-based index (TFQI); Data are reported as mean ± SD, median (1st–3rd quartile) or count (percentage).

The frequencies of normal thyroid function, TACITUS, Type 2 allostatic response and primary hypothyroidism were similar among survivors and non-survivors. However, thyrotoxicosis was significantly more prevalent in non-survivors compared to survivors (Table 4).

**Table 4:** Classes of thyroid function and total survival.

	Survivors (n = 69)	Non-survivors (all-cause fatality) (n = 38)	p
Normal thyroid function	21 (30%)	5 (13%)	0.079
TACITUS syndrome	26 (38%)	19 (50%)	0.087
Type 2 allostatic response	10 (14%)	2 (5%)	1.00
Thyrotoxicosis	10 (14%)	11 (29%)	0.038
Primary hypothyroidism	2 (3%)	1 (3%)	1.00

TACITUS, Thyroid Allostasis in Critical Illness, Tumors, Uremia, and Starvation.

#### Long-term fatality according to thyroid function

**Fig. 3:**
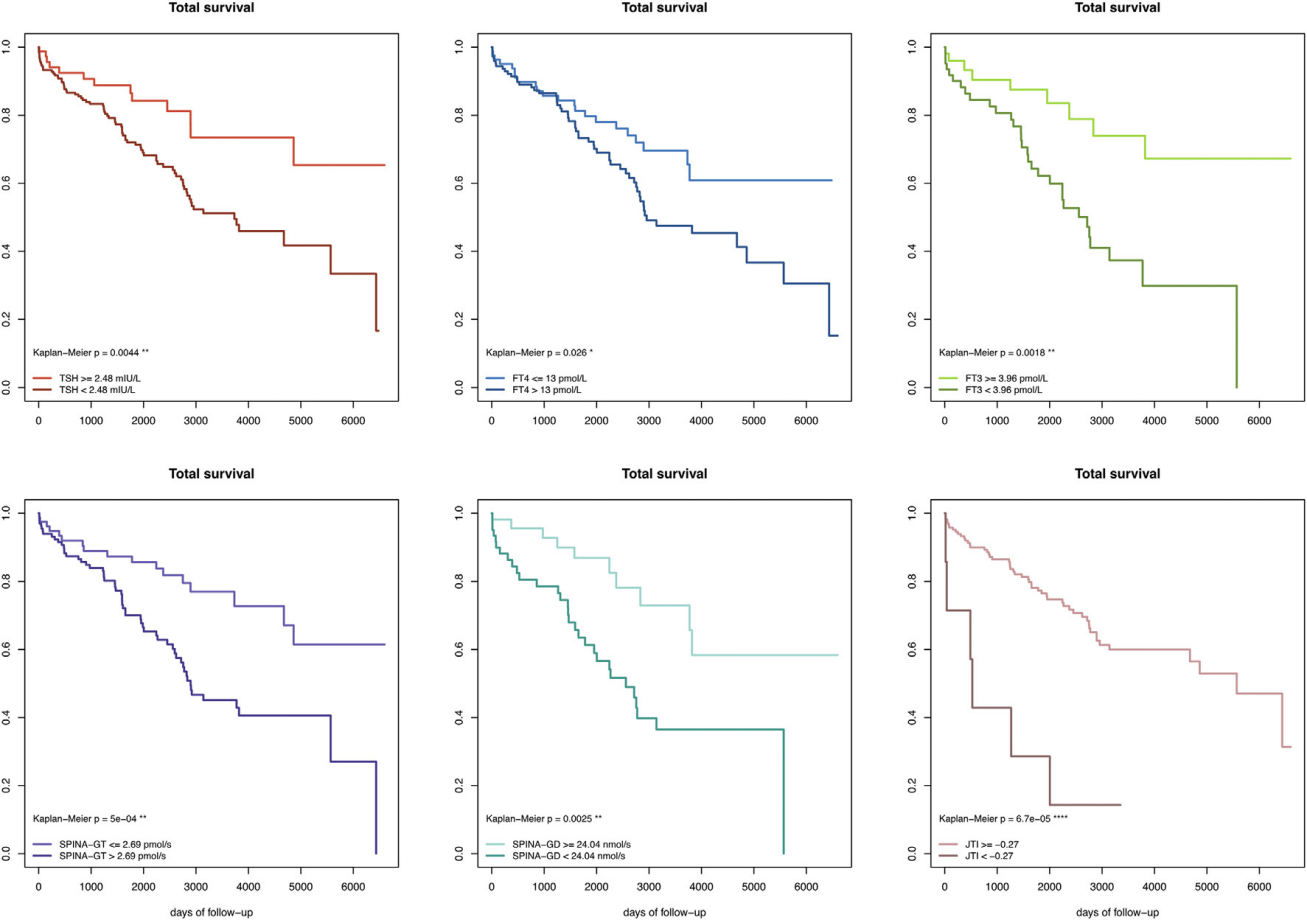
Kaplan–Meier survival curves based on cutoff values derived from phi statistics.

To find out optimal cut-off values of several thyroid parameters, we established an ROC analysis (Supplementary Fig. S2). Optimal cut-off values from phi statistics were for TSH 2.48 mlU/L, for FT4 13.0 pmol/L, for FT3 3.96 pmol/L, for SPINA−GT 2.69 pmol/s and for SPINA−GD 24.04 nmol/s. Optimal cut points based on Youden’s J were for TSH 2.08 mlU/L, for FT4 13.0 pmol/L, for FT3 3.96 pmol/L, for SPINA−GT 2.69 pmol/s and SPINA−GD 24.04 nmol/s. A TSH concentration ≥ 2.48 mIU/L was associated with significantly better survival compared to TSH < 2.48 mIU/L (p = 0.004 [log-rank]). FT4 ≤ 13 pmol/L was associated with a better survival compared to FT4 < 13 pmol/L (p = 0.03 [log-rank]) and FT3 ≥ 3.96 pmol/L predicted a better survival compared to FT3 < 3.96 pmol/L (p = 0.002 [log-rank]). Among calculated parameters, SPINA−GT ≤ 2.69 pmol/s was associated with better survival compared to SPINA−GT > 2.69 pmol/s (p < 0.0001 [log-rank]) and SPINA−GD ≥ 24.04 nmol/s predicted a better survival compared to SPINA−GD < 24.04 nmol/s (p = 0.003 [log-rank]) (Fig. 3 and Supplementary Fig. S3).

**Fig. 4:**
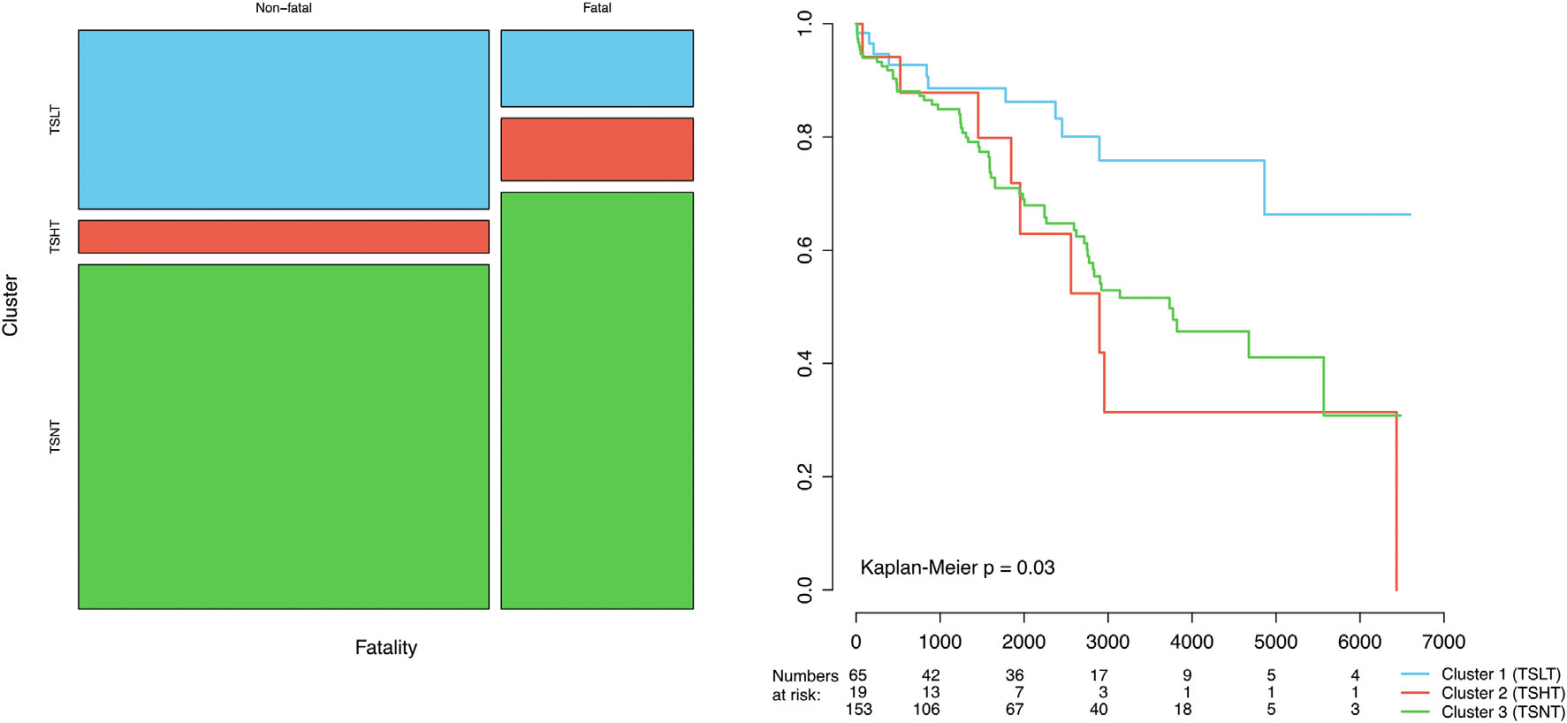
Association of clusters with total fatality (left) and time-course of survival (right).

Cluster 1 (TSLT) was associated with significantly better survival than clusters 2 (TSHT) and 3 (TSNT, p = 0.01 [Kaplan–Meier], Fig. 4).

#### Multivariable prediction model of survival

Using an adjusted minimal model (Table 5) for survival by multivariable cox regression concentrations of TSH and FT3, female sex and stressful triggers were associated with improved survival. Atrial fibrillation, neurological disease, physical stressful triggers, emotional stressful triggers, apical TTS form and low EF at admission were associated with a decreased survival rate.

**Table 5:** Univariable, adjusted minimal multivariable and hierarchical models from Cox regression for all-cause fatality.

	Univariable HR (95% CI)	p	Multivariable HR (95% CI)	p	Hierarchical HR (95% CI)	p
TSH (mIU/L)	0.87 (0.75–1.01)	0.067	0.79 (0.66–0.96)	0.015	0.88 (0.76–1.03)	0.10
FT4 (pmol/l)	1.00 (0.97–1.03)	0.94			0.99 (0.96–1.02)	0.62
FT3 (pmol/L)	0.76 (0.62–0.93)	0.0089	0.81 (0.65–0.99)	0.040	0.76 (0.62–0.94)	0.012
Age (years)	1.03 (1.02–1.05)	<0.0001				
Female sex	0.62 (0.40–0.96)	0.032	0.20 (0.09–0.44)	<0.0001		
Hypertension	0.97 (0.70–1.33)	0.84			0.97 (0.69–1.25)	0.85
Diabetes mellitus	1.45 (1.00–2.11)	0.051			1.52 (1.04–2.22)	0.032
Hypercholesterolemia	0.82 (0.60–1.13)	0.23			0.81 (0.58–1.20)	0.20
Smoking	0.80 (0.54–1.18)	0.26			0.21 (0.54–1.21)	0.30
Atrial fibrillation	2.09 (1.43–3.06)	<0.0001	4.16 (1.85–9.34)	<0.0001	2.18 (1.48–3.21)	<0.0001
Malignancy	1,72 (1.14–2.59)	0.0094			1.69 (1.11–2.57)	0.014
Neurological disease	1.43 (0.99–2.07)	0.058	6.63 (3.85–16.05)	<0.0001	1.48 (1.02–2.16)	0.041
Psychiatric disease	0.75 (0.40–1.37)	0.35			0.75 (0.40–1.39)	0.36
Stressful trigger	0.75 (0.55–1.03)	0.073	0.00 (0.00–0.01)	<0.0001	0.77 (0.56–1.06)	0.11
Physical trigger	1.45 (1.07–1.98)	0.018	253.04 (129.6–494.2)	<0.0001	1.44 (1.05–1.98)	0.024
Emotional trigger	0.58 (0.41–0.82)	0.0023	25.14 (11.14–56.72)	<0.0001	0.59 (0.41–0.85)	0.0042
Creatinine (μmol/L)	1.00 (1.00–1.01)	<0.0001			1.01 (1.00–1.01)	<0.0001
Apical ballooning	1.66 (1.12–2.47)	0.012	9.75 (3.59–26.44)	<0.0001	1.60 (1.07–2.39)	0.022
Midvent. ballooning	0.68 (0.44–1.04)	0.076			0.70 (0.45–1.09)	0.11
Basal ballooning	0.99 (0.37–2.68)	0.99			0.96 (0.35–2.62)	0.93
Focal ballooning	1.29 (0.32–5.19)	0.73			1.20 (0.29–4.93)	0.80
Initial EF (%)	0.96 (0.95–0.97)	<0.0001	0.93 (0.90–0.95)	<0.0001	0.96 (0.95–0.97)	<0.0001

TSH, Thyroxine stimulating hormone; FT3, free triiodothyronine; EF, left ventricular ejection fraction. For the hierarchical model age and sex were defined as shared frailty clusters.

### Secondary composite endpoint

Supplementary Table S6 provides an overview of the composite endpoint. Significantly more patients experiencing the secondary endpoint have atrial fibrillation, increased creatinine, reduced LVED, reduced SPINA-GD, and are on medication with diuretics, amiodarone, or thyroid replacement therapy.

No differences were found in TSH and FT4 concentrations between patients with and without cardiogenic shock. FT3 was significantly lower in persons with shock (2.4; 1.5–3.9 vs. 4.1; 3.2–4.8 pmol/L; p = 0.009 [Wilcoxon–Mann–Whitney test]; ratio of medians: 0.58; 0.35–0.93), but no association was found in Cox regression. Likewise, SPINA-GD (15.6; 9.0–24.5 vs. 23.4; 16.1–29.7 nmol/s; p = 0.03 [Wilcoxon–Mann–Whitney test]; ratio of medians: 0.67; 0.39–0.99) and Jostel’s TSH index (1.6; 0.6–2.7 vs. 2.3; 1.6–3.0; p = 0.03 [Wilcoxon–Mann–Whitney test]; ratio of medians: 0.69; 0.35–1.16) were lower in cases of cardiogenic shock (Supplementary Table S8). In univariable Cox analysis, increased creatinine concentration and lower LVEF were associated with the occurrence of shock. In the minimal model, low LVEF remained the only predictor of cardiogenic shock (Supplementary Table S5).

In the univariable and univariate analysis, FT4 concentration was increased in persons suffering from pulmonary oedema (Supplementary Table S8). TSH and FT3 concentrations as well as SPINA-GD and Jostel’s TSH index were decreased in subjects requiring catecholamine therapy (Supplementary Table S9). SPINA-GD was also significantly decreased in persons faced with stroke (Supplementary Table S12).

Thyroid function was not associated with other endpoints in single evaluation, including cardiopulmonary resuscitation (CPR), use of circulatory support (IABP or ECMO), invasive respiratory support, malignant arrhythmia and left ventricular thrombus formation (Supplementary Tables S10, S11, and S13). In the univariable Cox regression, the composite endpoint was predicted by creatinine concentration only, although the hazard ratio only minimally increased per μmol/L (Supplementary Table S14). The same pattern was observed in hierarchical analysis. No predictor could be identified in a multivariable model.

## Discussion

In this study, we analysed the distribution of endotypes of thyroid function in the GEIST multicentre registry and a potential association with the prognosis of TTS. The main findings were: (1) the prevalence of thyroid dysfunction is exceptionally high in TTS, and less than a quarter of included persons had normal thyroid homeostasis; (2) the patterns of thyroid function scatter in three clusters of low (TSLT), high (TSHT) and normal (TSNT) thyroid’s secretory capacity; (3) the TSHT cluster was associated with higher age, lower EF and higher frequency of physical triggers of TTS; (4) in non-survivors FT3 concentration and SPINA-GD were lower and SPINA-GT was higher compared to survivors, and the prevalence of thyrotoxicosis was higher in fatal cases; (5) low TSH and FT3 concentration, high FT4 concentration, low SPINA-GD and high SPINA-GT predicted long-term fatality in the Kaplan–Meier analysis; (6) the TSLT cluster predicts better long-term survival; (7) the FT3 concentration was lower in subjects affected by cardiogenic shock.

Our study presents a detailed multicentre analysis aimed at evaluating the clinical and prognostic significance of thyroid function in patients with TTS.

Both groups of primary thyrotoxicosis and type 2 allostatic load are marked by elevated concentrations of free T4 and fall into TSHT cluster. The high proportion of patients in TSHT cluster suggests a possible triggering mechanism for the development of TTS, which would be in accordance with our previous study and case reports observing an association between TTS and thyrotoxicosis.^12^^,^^19^^,^^29^ Interestingly, the rate of thyrotoxicosis in non-survivors among TTS is significantly higher compared to survivors. This finding is important to identify patients with TTS at higher risk.

Up to now, the distinct mechanisms by which thyrotoxicosis is able to elicit TTS remains poorly understood. Previous reports described TTS in thyrotoxicosis of different origins including true endogenous hyperthyroidism, destructive thyroiditis and factitious thyrotoxicosis.^12^^,^^20^, ^21^, ^22^, ^23^, ^24^

The sympatho-adrenergic system plays a pivotal role in the pathogenesis of TTS.^14^ Thyroid hormones are able to sensitize the heart for catecholamines by stimulating the expression of beta-adrenoceptors in cardiomyocytes, with subsequent positive inotropic and chronotropic effects.^26^^,^^28^^,^^37^, ^38^, ^39^ Therefore, an excess of thyroid hormones might potentiate the effects of catecholamines in the myocardial tissue, resulting in increased sensibility to stress events and heart stunning.^40^^,^^41^ Conversely, intracellular cAMP formed by activated beta-adrenoceptors is able to stimulate the expression of a number of genes including that of type 2 deiodinase (DIO2), which converts T4 to T3 and 3,5-T2, i.e., to more active thyroid hormones.^42^ Therefore, in the combination of stress with elevated concentration of T4 a positive feedback loop between catecholaminergic signaling and activation of thyroid hormones may arise, eventually igniting the transition to TTS.

The above-mentioned pattern of type 2 allostatic load includes higher FT4 concentrations despite non-suppressed TSH concentration. This pattern does not result from primary hyperthyroidism but marks the constellation of an elevated “set point” of the homeostatic system. A high set point is characterized by an increased or at least non-suppressed TSH concentration despite an elevated FT4 concentration.^43^ Temporarily or permanently elevated set points result from increased TSH secretion due to elevated hypothalamic TRH input. They have been described in conditions associated to type 2 allostatic load including obesity, acute psychosis, endurance training, adaptation to cold and post-traumatic stress disorder.^44^

The hypothalamic-pituitary-thyroid (HPT) axis acts as an adaptive, dynamic system, which in unstrained resting conditions operates as a homeostatic regulator, aiming at constant value control and maintaining serum concentrations of thyroid hormones in the vicinity of a fixed set point.^45^ However, in type 2 allostatic load resulting from an expected increase in energy demand, although the cumulative energy balance is still sufficient,^46^ elevations of the set point are common. This constellation is typical in psychosocial stress situations.^44^ Of note, psychosocial stress is known as the main trigger for TTS, and its effect may be mediated by altered thyroid hormone concentrations consistent with type 2 thyroid allostasis. Templin et al. demonstrated hypoconnectivity of central brain regions associated with autonomic functions and regulation of the limbic system in patients with TTS.^47^ The authors concluded that autonomic-limbic integration and particularly hypothalamic regions might play an important role in the pathophysiology of TTS. A possible linkage between type 2 thyroid allostasis and TTS could be given by an interaction between the so-called brain-heart axis and the HPT axis.

Abnormal thyroid hormone concentrations seem to be the rule rather than the exception in TTS as reported previously^30^ and this study confirmed this finding. It also reproduces a similar pattern of clusters as in our previous study.

In our cohort, the largest group of patients (42%) suffered from TACITUS syndrome, also referred to as non-thyroidal illness syndrome (NTIS), euthyroid sick syndrome (ESS) or low-T3-syndrome. It is well established that about 30% of hospitalized patients and more than 60% of patients affected by critical illness experience transient changes in serum concentrations of TSH and thyroid hormones.^44^ Characteristic patterns are low concentrations of FT3, impaired plasma protein binding of thyroid hormones and, in more severe cases, thyrotropic adaptation with a downward shift of the set point characterized by paradoxically low TSH levels in the presence of normal or even low concentrations of FT4. Over the previous decades, a plethora of studies confirmed in very different types of non-thyroidal illness that the presence of TACITUS syndrome predicts poor prognosis. In this study cohort, this condition did not indicate significantly higher fatality, although a trend to a worse prognosis could be observed. However, after performing a ROC analysis to determine thresholds dependent on thyroid hormone metabolic parameters we detected a considerable correlation to survival. In other words, all thyroid hormone metabolic parameters predict fatality. This finding is in accordance with epidemiologic observations demonstrating a U-shaped relationship between thyroid function and heart failure, severe arrhythmia and other adverse cardiovascular events.^28^^,^^48^^,^^49^

Our data underscore the importance of the thyroid–heart interaction and its impact on fatality among patients affected by TTS. This is even more interesting since historical investigations on endocrine characteristics of TTS focused only on the role of low estradiol levels and diabetes comorbidity and their impact on the prognosis of affected patients. Better biomarkers, which are associated with the prognosis of TTS are highly required, and thyroid hormones may be among them.

Several limitations have to be mentioned. This study is a sub-analysis of a cohort of patients included in the GEIST Registry. Of note, not in every patient all thyroid hormone concentrations of interest (TSH, FT3 and FT4) were recorded. Therefore, the sample size is not as high as we expected. Additionally, the fact that thyroid hormones were measured in a subgroup only may be a source of selection bias. This is a result of the observational nature of the registry design. Our additional analyses, where we compared persons with and without measurement of thyroid hormones, suggest, however, that this does not lead to a major distortion. It may still introduce sparse data bias, which may occur even in large datasets.^50^ This possibility is demonstrated by wide and potentially inflated confidence intervals and hazard ratio estimates for certain variables.

Reporting hazard ratios may raise problems due to potential change in HR over time and the generation of selection bias.^51^

The data were retrospectively analysed and originated from different laboratory analytic devices due to the multicentre design of this investigation. Therefore, the comparability of results obtained from different centers may be limited. Apart from the heterogeneity in the laboratory methods, the study design may introduce additional unmeasured confounding factors. We did not obtain systemic blood glucose levels. It is noteworthy that data suggest diabetes may have a protective effect in TTS.^52^

Furthermore, the etiology of thyroid dysfunction was not comprehensively documented. E.g., the group of primary thyrotoxicosis included Graves’ disease, toxic multinodular goitre and factitious thyrotoxicosis, but was in the majority of cases not characterized from a nosological perspective, and data from imaging studies or antibody determination were not available. The role of time series of thyroid hormone concentrations during the in-hospital stay could not be evaluated in the present analysis due to low sample size. This should be a topic of a future prospective study if enough data points can be obtained.

In summary, the present study demonstrates that thyroid hormones play a significant role in the evolution of TTS and its prognosis. It might, therefore, pave the way to novel therapeutic options. Innovative thyroid-derived biomarkers may help to identify high-risk patients with TTS and promote a personalized and preventive use of several drugs to reduce all-cause fatality.

## Contributors

All authors take responsibility for the content of the manuscript. AA, JWD, IA, IE contributed to the conception and design of the study. AA, JWD, MCF, IA, IE, AM analysed and interpreted the patient data. AA, JWD, FS, TS, IE have directly accessed and verified the underlying data in the manuscript. AA, JWD, IE performed the statistical analyses. AA, JWD, IA, IE were major contributors in writing the manuscript. All authors contributed to data collection. All authors read and approved the final manuscript.

## Data sharing statement

The datasets used for the analysis in the current study and source code for evaluation are available from the corresponding author upon reasonable request.

## Declaration of interests

JWD received funding and personal fees from Novo Nordisk, VitalAire, Abbott, Medtronic, Oviva, Egetis Therapeutics, myhomecare, aidhere, Ascensia Diabetes Care, Sanofi-Henning, Hexal AG, Bristol-Myers Squibb, and Pfizer, and is the co-owner of the intellectual property rights for the patent “System and Method for Deriving Parameters for Homeostatic Feedback Control of an Individual” (Singapore Institute for Clinical Sciences, Biomedical Sciences Institutes, Application Number 201208940-5, WIPO number WO/2014/088516). IE received funding from Else-Kröner-Fresenius Foundation (No. 2022_EKES.48) and Innovationforum (No. IF-034-22). All other authors declare no conflict of interest.
