# Supplementary material for: Takotsubo syndrome outcomes predicted by thyroid hormone signature: insights from cluster analysis of a multicentre registry
Source: eBioMedicine. 2024 Mar 18;102:105063. doi: 10.1016/j.ebiom.2024.105063 (PMC10963195; doi:10.1016/j.ebiom.2024.105063)
Supplement: Supplementary Figs. S1–S3 and Tables S1–S14 [file mmc1.pdf]

eBioMedicine
Part of THE LANCET Discovery Science

Supplementary Appendix accompanying the manuscript „Takotsubo Syndrome Outcomes Predicted by Thyroid Hormone Signature: Insights from Cluster Analysis of a Multicenter Registry"

Assem Aweimer, Johannes W. Dietrich, Francesco Santoro, Mireia Camins Fàbregas, Andreas Mügge, Iván J Núñez-Gil, Ravi Vazirani, Oscar Vedia, Toni Pätz, Ilaria Ragnatela, Luca Arcari, Massimo Volpe, Miguel Corbì-Pascual, Manuel Martinez-Selles, Manuel Almendro-Delia, Alessandro Sionis, Aitor Uribarri, Holger Thiele, Natale Daniele Brunetti, Ingo Eitel, Thomas Stiermaier, Nazha Hamdani, Mohammad Abumayyaleh, Ibrahim Akin, Ibrahim El-Battrawy

Supplementary Methods

Calculations

SPINA-GT (thyroid's secretory capacity) was calculated with

$$\hat{G}_T = \frac{\beta_T (D_T + [TSH])(1 + K_{41}[TBG] + K_{42}[TTR])[FT_4]}{\alpha_T [TSH]}$$

from steady-state concentrations of thyrotropin (TSH) and free thyroxine (FT4) and constant parameters for plasma protein binding, distribution and elimination, as previously described[1].

Similarly, SPINA-GD (sum activity of step-up deiodinases) was calculated with

$$\hat{G}_D = \frac{\beta_{31}(K_{M1} + [FT_4])(1 + K_{30}[TBG])[FT_3]}{\alpha_{31}[FT_4]}$$

from concentrations of FT3, FT4 and constants for the kinetics of hormones[1].

Parameters for the central function of the feedback loop (the so-called set point) included Jostel's TSH index[2]

$$TSHI = \ln([TSH]) + \beta[FT_4],$$

the thyrotroph thyroid hormone sensitivity index (TTSI)[3,4]

$$TTSI = \frac{100[TSH][FT_4]}{l_U}$$

and the thyroid Feedback Quantile-based Index (TFQI)[5]

$$TFQI = F_{FT4}[FT_4] - (1 - F_{TSH}[TSH]).$$

Table S1: Parameters for calculation[1]

Parameter	Explanation	Value
α_T	Dilution factor for thyroxine	0.1 l^{-1}
β_T	Clearance exponent for T4	$1.1 \cdot 10^{-6}$ sec^{-1}
D_T	EC_{50} for TSH	2.75 mIU/L
K_{41}	Dissociation constant of T4 at thyroxine binding globulin	$2 \cdot 10^{10}$ L/mol
K_{42}	Dissociation constant of T4 at transthyretin	$2 \cdot 10^{8}$ L/mol
α_{31}	Dilution factor for triiodothyronine	0.026 l^{-1}
β_{31}	Clearance exponent for T3	$8 \cdot 10^{-6}$ sec^{-1}
K_{M1}	Dissociation constant of type 1 deiodinase	500 nmol/L
K_{30}	Dissociation constant of T3 at thyroxine binding globulin	$2 \cdot 10^{9}$ L/mol
[*TBG*]	Standard concentration of thyroxine binding globulin	300 nmol/L
[*TTR*]	Standard transthyretin concentration	4.5 µmol/L
β	Correction coefficient of logarithmic model	0.1345

Power analysis and sample size estimation

Assuming a drop of FT3 concentration by 0.5 pmol/L a sample size of 90 per group is necessary to achieve a power of 0.9. 70 per group is necessary to arrive at a power of 0.8 (Fig. S1).

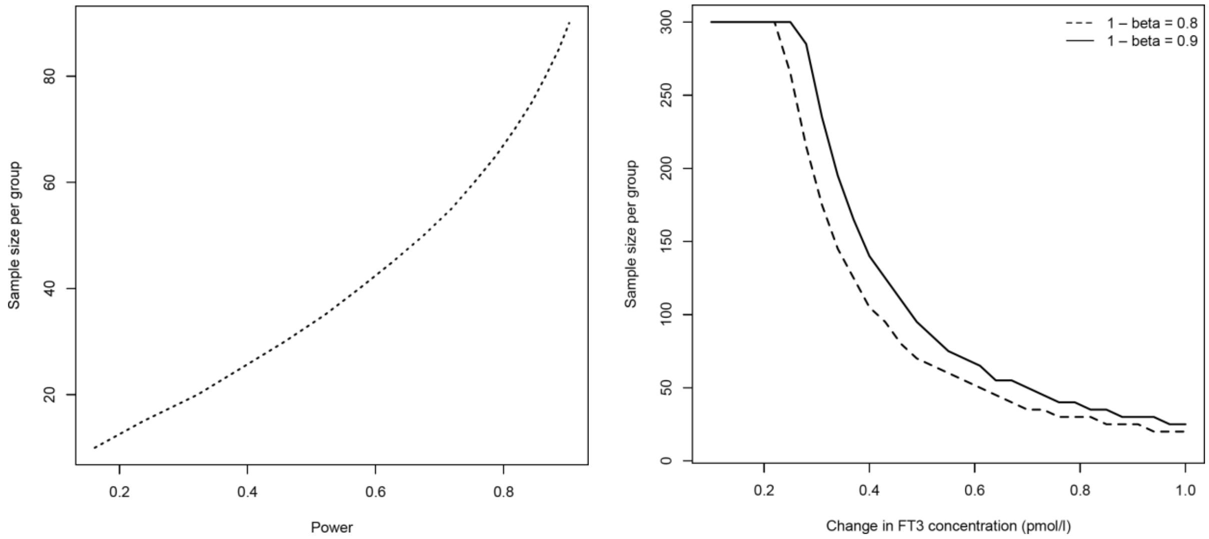

Figure S1: Sample size depending on power for a given drop of FT3 concentration by 0.5 pmol/L (left) and depending on change in FT3 concentration (right). The plots are derived from Monte Carlo simulations of the empirical power of a Wilcoxon rank sum test with the R package MKpower[6].

Supplementary Results

Table S2: Characteristics of the study population concerning the measurement of thyroid function. Data are reported as mean ± SD, median (1st–3rd quartile) or count (percentage). Data are reported as mean ± SD, median (1st–3rd quartile) or count (percentage).

	TSH measured (n = 411)	TSH not measured (n = 279)	p	FT4 measured (n = 289)	FT4 not measured (n = 401)	p	FT3 measured (n = 169)	FT3 not measured (n = 521)	p
Survivors (%)	253 (72.5%)	192 (72.2%)	1.00	172 (68.3%)	273 (75,2%)	0.071	91 (68.9%)	354 (73.3%)	0.38
Age (years)	69.9 ± 12.7	70.3 ± 12.4	0.61	70.6 ± 12.6	69.7 ± 12.6	0.32	70.1 ± 12.6	70.1 ± 12.6	0.96
Female (%)	364 (88.6%)	247 (88.5%)	1.00	257 (88.9%)	354 (88.3%)	0.89	147 (87.0%)	464 (89.1%)	0.55
Male (%)	47 (11.4%)	32 (11,5%)		32 (11.1%)	47 (11.7%)		22 (13.0%)	57 (10.9%)	
BMI (kg/m^2)	24.4 ± 5.3	24.8 ± 4.6	0.17	23.9 ± 5.9	24.8 ± 4.7	0.036	24.2 ± 4.7	24.7 ± 6.2	0.13
Hypertension (%)	257 (62.5%)	188 (67.4%)	0.22	187 (64.7%)	258 (64.3%)	0.99	107 (63.3%)	338 (64.9%)	0.78
Diabetes mellitus (%)	76 (18.5%)	44 (15.8%)	0.41	57 (19.7%)	63 (15.7%)	0.20	32 (18.9%)	88 (16.9%)	0.62
Atrial fibrillation (%)	71 (17.3%)	41 (14.7%)	0.43	50 (17.3%)	62 (15.5%)	0.59	37 (21.9%)	75 (14.4%)	0.029
Hypercholesterolemia (%)	119 (29.0%)	111 (39.8%)	0.0039	105 (36.3%)	125 (31.2%)	0.18	56 (33.1%)	147 (29.8%)	1.00
Smoking (%)	87 (21.2%)	63 (22.6%)	0.73	49 (17.0%)	101 (25.2%)	0.013	36 (21.3%)	114 (21.9%)	0.96
Coronary artery disease (%)	60 (14.6%)	23 (8.2%)	0.016	39 (13.5%)	44 (11.0%)	0.38	29 (17.2%)	54 (10.4%)	0.026
Malignancy (%)	40 (9.8%)	46 (16.5%)	0.012	28 (9.7%)	58 (14.5%)	0.079	20 (11.8%)	66 (12.7%)	0.88
Neurological disease (%)	79 (17.6%)	49 (19.2%)	0.65	63 (21.8%)	65 (16.2%)	0.078	37 (21.9%)	91 (17.5%)	0.24
Psychiatric disease (%)	37 (9.0%)	22 (7.9%)	0.71	26 (9.0%)	33 (8.2%)	0.83	15 (8.9%)	44 (8.4%)	0.99
Stressful trigger (%)	285 (69.3%)	180 (64.5%)	0.21	203 (70.2%)	262 (65.3%)	0.20	120 (71.0%)	345 (66.2%)	0.29
Physical trigger (%)	141 (34.3%)	100 (35.8%)	0.74	98 (33.9%)	143 (35.7%)	0.69	60 (35.5%)	181 (34.7%)	0.93
Emotional trigger (%)	146 (35.5%)	85 (30.5%)	0.19	107 (37.0%)	124 (30.9%)	0.11	58 (34.3%)	173 (33.2%)	0.86
Creatinine concentration (µmol/L)	81.3 (67.0–102.5)	79.1 (66.7–101.4)	0.97	85.7 (70,7–107.4)	77.8 (64.0–99.0)	0.015	83.9 (70.7–105.2)	79.1 (64.0–101.3)	0.062
Apical ballooning (%)	294 (71.5%)	200 (71.7%)	0.71	218 (75.4%)	276 (68.8%)	0.054	130 (76.9%)	364 (71.2%)	0.048
Midventricular ballooning (%)	101 (21.1%)	59 (24.6%)	0.34	54 (18.7%)	106 (26.4%)	0.022	34 (20.1%)	126 (24.2%)	0.33
Basal ballooning (%)	5 (1.2%)	9 (3.2%)	0.096	5 (1.7%)	4 (1.0%)	0.42	3 (1.8%)	11 (2.1%)	1.00

Initial EF (%)	42.2 ± 13.6	43.8 ± 13.9	0.10	43.9 ± 13.2	42.0 ± 14.1	0.22	41.9 ± 13.0	43.1 ± 14.0	0.20
Antihypertensive drugs (%)	344 (83.7%)	234 (83.9%)	1.00	234 (81.0%)	344 (85,8%)	0.11	143 (84.6%)	435 (83.5%)	0.82
ACE-I or ARB (%)	253 (61.5%)	198 (71.0%)	0.014	162 (56.1%)	289 (72.1%)	<0.0001	95 (56.2%)	336 (68.3%)	0.0053
Beta blockers (%)	274 (66.7%)	158 (66.3%)	0.99	178 (61.6%)	281 (70.1%)	0.025	112 (66.3%)	347 (66.6%)	1.00
Diuretics (%)	152 (37.0%)	95 (33.7%)	0.42	100 (34.6%)	146 (36.4%)	0.68	73 (43.2%)	173 (31.1%)	0.024
Aldosterone antagonists (%)	25 (6.1%)	17 (6.1%)	1.00	9 (3.1%)	33 (8.2%)	0.0090	8 (4.7%)	34 (6.5%)	0.51
Statins (%)	184 (44.8%)	137 (49.1%)	0.30	136 (47.1%)	185 (46.1%)	0.87	79 (46.7%)	242 (46.4%)	1.00
Amiodarone (%)	11 (2.7%)	6 (2.2%)	0.85	9 (3.1%)	8 (2.0%)	0.49	5 (3.0%)	12 (2.3%)	0.58
Anticoagulation (%)	136 (33.7%)	60 (21.7%)	0.0009	81 (28.3%)	115 (29.2%)	0.87	57 (34.1%)	139 (27.1%	0.10
Antidiabetic medication (%)	49 (14.2%)	16 (10.1%)	0.26	38 (13.8%)	27 (11.9%)	0.62	23 (14.7%)	42 (12.1%)	0.50
Thyroid medication (%)	72 (17.6%)	13 (7.7%)	0.0038	54 (18.7%)	31 (10.7%)	0.0098	39 (23.1%)	46 (11.2%)	0.00043
Levothyroxine (%)	41 (10.0%)	2 (0.7%)	<0.0001	23 (8.0%)	20 (5.0%)	0.15	19 (11.2%)	24 (4.6%)	0.0035
Perchlorate (%)	8 (1.9%)	1 (0.4%)	0.092	8 (2.8%)	1 (0.2%)	0.0051	6 (3.6%)	3 (0.6%)	0.010
Iodine (%)	2 (0.5%)	0 (0%)	0.52	1 (0.3%)	1 (0.3%)	1.00	1 (0.6%)	1 (0.2%)	0.43
Antithyroid agents (%)	1 (0.2%)	0 (0%)	1.00	1 (0.3%)	0 (0%)	0.42	1 (0.6%)	0 (0%)	0.24

Table S3: Basic characteristics of the full population of the GEIST registry, including persons that did not undergo thyroid function testing.

	**Survivors (n = 445)**	**Non-survivors (all-cause fatality) (n = 170)**	**p**
Age (years)	68.1 ± 12.7	73.5 ± 11.2	<0.0001
Female (%)	400 (89.9%)	146 (89.9%)	0.21
Male (%)	34 (10.1%)	24 (10.1%)	
BMI (kg/m^2)	24.6 ± 5.1	24.2 ± 5.2	0.58
Hypertension (%)	273 (61.3%)	112 (65.9%)	0.34
Diabetes mellitus (%)	63 (14.2%)	35 (20.6%)	0.068
Atrial fibrillation (%)	55 (12.4%)	34 (20.0%)	0.023
Hypercholesterolemia (%)	140 (31.5%)	57 (33.5%)	0.69
Smoking (%)	102 (22.9%)	32 (18.8%)	0.32
Coronary artery disease (%)	46 (10.3%)	22 (12.9%)	0.44
Malignancy (%)	49 (11.0%)	28 (16.5%)	0.090
Neurological disease (%)	78 (17.5%)	37 (21.8%)	0.28
Psychiatric disease (%)	44 (9.9%)	11 (6.5%)	0.24
Stressful trigger (%)	314 (70.6%)	102 (60.0%)	0.016
Physical trigger (%)	143 (32.1%)	69 (40.6%)	0.060
Emotional trigger (%)	174 (39.1%)	41 (24.1%)	0.0001
Creatinine concentration (μmol/L)	76.0 (66.0–89.3)	105.2 (81.3–147.6)	<0.0001
Apical ballooning (%)	303 (68.1%)	139 (81.8%)	0.0011
Midventricular ballooning (%)	115 (25.8%)	25 (14.7%)	0.0045
Basal ballooning (%)	9 (2.0%)	4 (2.4%)	1.00
Initial EF (%)	44.4 ± 13.6	39.5 ± 14.3	<0.0001
Antihypertensive drugs (%)	375 (84.3%)	130 (76.5%)	0.032
ACE-I or ARB (%)	304 (68.3%)	93 (54.7%)	0.0022
Beta blockers (%)	301 (67.6%)	94 (55.3%)	0.0057
Diuretics (%)	130 (29.2%)	69 (40.6%)	0.0094
Aldosterone antagonists (%)	27 (6.1%)	6 (3.5%)	0.29
Statins (%)	204 (42.8%)	72 (42.4%)	0.49
Amiodarone (%)	12 (2.7%)	4 (2.4%)	1.00
Anticoagulation (%)	124 (28.2%)	49 (29.0%)	0.93
Antidiabetic medication (%)	33 (10.0%	18 (13.2%)	0.39
Thyroid medication (%)	57 (15.6%)	19 (13.2%)	0.58
Levothyroxine (%)	32 (7.2%)	7 (4.1%)	0.22
Perchlorate (%)	6 (1.3%)	3 (1.8%)	0.71
Iodine (%)	2 (0.4%)	0 (0%)	1.00
Antithyroid agents (%)	1 (0.2%)	0 (0%)	1.00

Table S4: Markers of thyroid function in the subpopulation that received a full panel of thyroid investigation.

	**Survivors (n = 90)**	**Non-survivors (all-cause fatality; n = 41)**	**p**
TSH (mIU/L)	1.3 (0.5–3.3)	1.0 (0.3–1.7)	0.11
FT4 (pmol/L)	14.5 (12.5–17.1)	14.3 (13.1–18.4)	0.44
FT3 (pmol/L)	4.1 (3.1–4.6)	3.3 (1.5–3.9)	0.015
SPINA-GT (pmol/s)	2.9 (2.0–6.7)	4.2 (2.8–10.2)	0.12
SPINA-GD (nmol/s)	26.2 (16.4–30.3)	18.9 (11.3–25.0)	0.0066
JTI	2.2 (1.4–3.0)	2.0 (1.1–2.5)	0.073
TTSI	62.0 (23.6–141.0)	49.8 (17.6–85.6)	0.12
TFQI	0.2 (0.0–0.6)	0.2 (0.0–0.5)	0.17

Table S5: Univariable Cox regression and adjusted minimal model for cardiogenic shock.

	**Univariable HR (95% CI)**	**p**	**Minimal model HR (95% CI)**	**p**
TSH (mIU/L)	1.03 (0.85–1.25)	0.76		
FT4 (pmol/l)	0.99 (0.92–1.06)	0.77		
FT3 (pmol/L)	1.11 (0.92–1.35)	0.29		
Age (years)	0.98 (0.96–1.00)	0.057		
Female sex	0.55 (0.27–1.10)	0.093		
Hypertension	0.90 (0.50–1.61)	0.72		
Diabetes mellitus	1.02 (0.55–1.93)	0.96		
Hypercholesterolemia	0.69 (0.37–1.28)	0.24		
Smoking	0.94 (0.49–1.82)	0.86		
Atrial fibrillation	1.02 (0.55–1.87)	0.96		
Malignancy	0.63 (0.27–1.47)	0.29		
Neurological disease	1.31 (0.71–2.39)	0.38		
Psychiatric disease	0.78 (0.30–2.01)	0.61		
Stressful trigger	1.04 (0.59–1.84)	0.89		
Physical trigger	1.38 (0.80–2.38)	0.25		
Emotional trigger	0.80 (0.40–1.63)	0.54		
Creatinine (µmol/L)	1.00 (1.00–1.01)	0.049		
Apical ballooning	1.02 (0.52–1.99)	0.96		
Midvent. ballooning	1.58 (0.87–2.87)	0.14		
Basal ballooning	$1.09 \cdot 10^{-7}$ (0–∞)	1.00		
Focal ballooning	$1.09 \cdot 10^{-7}$ (0–∞)	1.00		
Initial EF (%)	0.96 (0.94–0.98)	<0.0001	0.96 (0.94–0.98)	<0.0001

Table S6: Comparison of subjects meeting and not meeting the composite endpoint of in-hospital complications (arrhythmias, thromboembolic events or stroke, pulmonary congestion, cardiogenic shock using catecholamine or assist device).

	**Not meeting the composite endpoint (n = 479)**	**Meeting the composite endpoint (n = 211)**	**p**
Age (years)	70.0 ± 12.4	70.3 ± 13.1	0.53
Female (%)	433 (90.4%)	178 (84.4%)	0.030
Male (%)	46 (9.6%)	33 (15.6%)	
BMI (kg/m^2)	24.5 ± 4.7	25.0 ± 5.9	0.85
Hypertension (%)	306 (63.9%)	139 (65.9%)	0.68
Diabetes mellitus (%)	79 (16.5%)	41 (19.4%)	0.41
Atrial fibrillation (%)	60 (12.5%)	52 (24.6%)	<0.0001
Hypercholesterolemia (%)	151 (31.5%)	79 (37.4%	0.15
Smoking (%)	111 (29.3%)	39 (18.5%)	0.20
Coronary artery disease (%)	57 (11.9%)	26 (12.3%)	0.98
Malignancy (%)	56 (11.7%)	30 (14.2%)	0.42
Neurological disease (%)	80 (16.7%)	48 (22.7%)	0.076
Psychiatric disease (%)	40 (8.4%)	19 (9.0%)	0.89
Stressful trigger (%)	321 (67.0%)	144 (68.2%	0.82
Physical trigger (%)	158 (33.0%)	83 (39.3%)	0.13
Emotional trigger (%)	166 (34.7%)	65 (30.8%)	0.37
Creatinine concentration (µmol/L)	75.1 (64.4–95.3)	90.2 (73.2–120.2)	<0.0001
Apical ballooning (%)	331 (69.1%)	163 (77.3%)	0.071
Midventricular ballooning (%)	116 (24.2%)	44 (20.9%)	0.39
Basal ballooning (%)	10 (2.9%)	4 (1.9%)	1.00
Focal ballooning (%)	9 (1.9%)	0 (0.0%)	0.10
Initial EF (%)	44.1 ± 13.6	40.0 ± 13.7	<0.0001
Antihypertensive drugs (%)	405 (84.5%)	173 (82.0%)	0.47
ACE-I or ARB (%)	334 (69.7%)	117 (55.5%)	<0.0001
Beta blockers (%)	328 (68.5%)	131 (62.1%)	0.12
Diuretics (%)	155 (32.4%)	91 (43.1%)	0.008
Aldosterone antagonists (%)	28 (5.8%)	14 (6.6%)	0.82
Statins (%)	228 (47.6%)	93 (44.1%)	0.44
Amiodarone (%)	3 (0.6%)	14 (6.6%)	<0.0001
Anticoagulation (%)	118 (25.0%)	78 (37.5%)	0.0013
Antidiabetic medication (%)	38 (11.5%)	27 (15.7%)	0.23
Thyroid medication (%)	65 (17.0%)	20 (10.3%)	0.042
Levothyroxine (%)	33 (6.9%)	10 (4.7%)	0.37
Perchlorate (%)	4 (0.8%)	5 (2.4%)	0.20
Iodine (%)	2 (0.4%)	0 (0.0%)	0.86
Antithyroid agents (%)	1 (0.2%)	0 (0.0%)	1.00
TSH (mIU/L)	1.4 (0.7–2.5)	1.3 (0.7–2.6)	0.77
FT4 (pmol/L)	14.1 (12.3–16.7)	15.3 (12.8–18.4)	0.060
FT3 (pmol/L)	4.2 (3.2–4.8)	3.9 (2.0–4.7)	0.093
SPINA-GT (pmol/s)	3.2 (2.3–6.5)	3.8 (2.4–6.6)	0.51
SPINA-GD (nmol/s)	23.8 (18.0–30.1)	20.1 (11.7–27.5)	0.026
JTI	2.1 (1.5–2.9)	2.5 (1.5–3.2)	0.29
TTSI	60.6 (27.5–108.5)	67.2 (28.0–148.3)	0.35
TFQI	0.3 (0.0–0.6)	9.3 (0.0–0.6)	0.26

Table S7: Comparing subjects with and without cardiogenic shock

	**No shock (n = 634)**	**Shock (n = 55)**	**p**
Age (years)	70.3 ± 12.6	67.8 ± 12.3	0.12
Female (%)	565 (89.1%)	45 (81.8%)	0.16
Male (%)	69 (10.9%)	10 (18.2%)	
BMI (kg/m^2)	24.5 ± 4.7	25.9 ± 8.0	0.67
Hypertension (%)	407 (64.2%)	38 (69.1%)	0.56
Diabetes mellitus (%)	108 (17.0%)	12 (21.8%)	0.48
Atrial fibrillation (%)	96 (15.1%)	16 (29.1%)	0.012
Hypercholesterolemia (%)	216 (34.1%)	14 (25.5%)	0.25
Smoking (%)	137 (21.6%)	13 (23.6%)	0.86
Coronary artery disease (%)	74 (11.7%)	9 (16.4%)	0.42
Malignancy (%)	80 (12.6%)	6 (10.9%)	0.88
Neurological disease (%)	112 (17.7%)	15 (27.3%)	0.11
Psychiatric disease (%)	54 (8.5%)	5 (9.1%)	1.00
Stressful trigger (%)	428 (67.5%)	36 (65.5%)	0.87
Physical trigger (%)	213 (33.6%)	28 (50.9%)	0.015
Emotional trigger (%)	220 (34.7%)	10 (18.2%)	0.019
Creatinine conc. (µmol/L)	79.6 (66.2–99.7)	95.5 (83.1–145.9)	0.00016
Apical ballooning (%)	450 (71.0%)	44 (80%)	0.34
Midventricular ballooning (%)	144 (22.7%)	15 (27.3%)	0.55
Basal ballooning (%)	14 (2.2%)	0 (0%)	0.54
Focal ballooning (%)	9 (1.4%)	0 (0%)	0.79
Initial EF (%)	43.7 ± 13.5	32.5 ± 12.5	<0.0001
Antihypertensive drugs (%)	539 (72.1%)	25 (45.5%)	0.0039
ACE-I or ARB (%)	425 (67.0%)	25 (45.5%)	0.0021
Beta blockers (%)	433 (68.3%)	25 (45.5$)	0.00099
Diuretics (%)	225 (35.5%)	21 (38.2%)	0.80
Aldosterone antagonists (%)	41 (6.5%)	1 (1.8%)	0.28
Statins (%)	300 (47.3%)	21 (38.2%)	0.25
Amiodarone (%)	12 (1.9%)	5 (9.1%)	0.0044
Anticoagulation (%)	174 (27.8%)	21 (38.9%)	0.12
Antidiabetic medication (%)	60 (13.0%)	5 (12.2%)	1.00
Thyroid medication (%)	78 (14.7%)	7 (15.2%)	1.00
Levothyroxine (%)	41 (6.5%)	2 (3.6%)	0.59
Perchlorate (%)	6 (1.0%)	3 (5.5%)	0.029
Iodine (%)	2 (0.3%)	0 (0%)	1.00
Antithyroid agents (%)	1 (0.2%)	0 (0%)	1.00
TSH (mIU/L)	1.4 (0.7–2.5)	1.3 (0.3–2.7)	0.52
FT4 (pmol/L)	14.5 (12.5–17.4)	14.2 (12.5–16.3)	0.70
FT3 (pmol/L)	4.1 (3.2–4.8)	2.4 (1.5–3.9)	0.088
SPINA-GT (pmol/s)	3.3 (2.3–6.3)	4.0 (1.9–14.1)	0.58
SPINA-GD (nmol/s)	23.4 (16.1–29.7)	15.6 (9.–24.5)	0.032
JTI	2.3 (1.6–3.0)	1.4 (0.6–2.7)	0.030
TTSI	66.5 (30.8–116.7)	36.1 (11.7–115.0)	0.086
TFQI	0.27 (0.04–0.60)	0.09 (0.00–0.59)	0.14

Table S8: Comparing subjects with and without pulmonary oedema

	**No pulmonary oedema (n = 669)**	**Pulmonary oedema (n = 21)**	**p**
Age (years)	69.9 ± 12.7	75.8 ± 9.1	0.041
Female (%)	594 (88.8%)	17 (80.9%)	0.45
Male (%)	75 (11.2%)	4 (19.1%)	
BMI (kg/m^2)	24.6 ± 5.1	24.3 ± 3.7	0.91
Hypertension (%)	430 (64.3%)	15 (71.4%)	0.66
Diabetes mellitus (%)	114 (17.0%)	6 (28.6%)	0.28
Atrial fibrillation (%)	105 (15.7%)	7 (33.3%)	0.063
Hypercholesterolemia (%)	224 (33.5%)	6 (28.6%)	0.81
Smoking (%)	145 (21.7%)	5 (23.8%)	1.00
Coronary artery disease (%)	77 (11.5%)	6 (28.6%	0.043
Malignancy (%)	82 (12.3%)	4 (19.0%)	0.55
Neurological disease (%)	120 (17.9%)	8 (38.1%)	0.040
Psychiatric disease (%)	56 (8.4%)	3 (14.3%)	0.58
Stressful trigger (%)	453 (67.7%)	12 (57.1%)	0.43
Physical trigger (%)	233 (34.8%)	8 (38.1%)	0.94
Emotional trigger (%)	228 (34.1%)	3 (14.3%)	0.10
Creatinine concentration (µmol/L)	79.6 (66.3–102.5)	88.0 (70.8–108.0)	0.22
Apical ballooning (%)	479 (71.6%)	15 (71.4%)	0.98
Midventricular ballooning (%)	154 (23.0%)	6 (28.6%)	0.74
Basal ballooning (%)	14 (2.1%)	0 (0%)	1.00
Focal ballooning (%)	9 (1.3%)	0 (0%)	1.00
Initial EF (%)	43.1 ± 12.7	35.6 ± 12.8	0.0046
Antihypertensive drugs (%)	557 (83.3%)	21 (100%)	0.080
ACE-I or ARB (%)	437 (65.3%)	19 (90.5%)	1.00
Beta blockers (%)	440 (65.8%)	19 (90.5%)	0.033
Diuretics (%)	230 (34.3%)	16 (76.2%)	<0.0001
Aldosterone antagonists (%)	36 (5.4%)	6 (28.6%)	<0.0001
Statins (%)	310 (46.3%)	11 (52.4%)	0.75
Amiodarone (%)	16 (2.4%)	1 (4.8%)	1.00
Anticoagulation (%)	188 (28.5%)	8 (38.1%)	0.48
Antidiabetic medication (%)	61 (12.3%)	4 (44.4%)	0.019
Thyroid medication (%)	85 (15.2%)	0 (0%)	0.15
Levothyroxine (%)	43 (6.4%	0 (0%)	0.46
Perchlorate (%)	9 (1.3%)	0 (0%)	1.00
Iodine (%)	2 (0.3%)	0 (0%)	1.00
Antithyroid agents (%)	1 (0.1%)	0 (0%)	1.00
TSH (mIU/L)	1.4 (0.7–2.5)	1.8 (0.7–2.6)	0.82
FT4 (pmol/L)	14.3 812.5–17.2)	17.6 (16.7–18.8)	0.035
FT3 (pmol/L)	4.1 (3.1–4.7)	3.8 (2.8–4.4)	0.51
SPINA-GT (pmol/s)	3.3 (2.3–6.6)	4.0 (2.8–6.0)	0.60
SPINA-GD (nmol/s)	23.2 (15.5–29.6)	18.8 (12.2–22.5)	0.14
JTI	2.2 (1.5–3.0)	2.5 (2.1–3.7)	0.26
TTSI	64.2 (27.3–112.4)	66.5 (45.2–186.0)	0.39
TFQI	0.27 (0.03–0.59)	0.34 (0.15–0.77)	0.42

Table S9: Comparing subjects with and without catecholamine therapy

	**No pulmonary catecholamine therapy (n = 629)**	**Catecholamine therapy (n = 61)**	**p**
Age (years)	70.4 ± 12.3	66.0 ± 14.6	0.031
Female (%)	599 (89.5%)	52 (85.2%)	0.52
Male (%)	30 (10.5%)	9 (14.8%)	
BMI (kg/m^2)	24.5 ± 4.7	25.7 ± 8.3	1.00
Hypertension (%)	408 (64.9%)	37 (60.7%)	0.61
Diabetes mellitus (%)	107 (17.0%)	13 (21.3%)	0.50
Atrial fibrillation (%)	99 (15.7%)	13 (21.3%)	0.34
Hypercholesterolemia (%)	213 (33.9%)	17 (27.9%)	0.42
Smoking (%)	134 (21.3%)	16 (26.2%)	0.47
Coronary artery disease (%)	73 (11.6%)	10 (16.4%)	0.37
Malignancy (%)	80 (12.7%)	6 (9.8%)	0.65
Neurological disease (%)	112 (17.8%)	16 (26.2%)	0.15
Psychiatric disease (%)	54 (8.6%)	5 (8.2%)	1.00
Stressful trigger (%)	424 (67.4%)	41 (67.2%)	1.00
Physical trigger (%)	213 (33.9%)	28 (45.9%)	0.081
Emotional trigger (%)	217 (34.5%)	14 (23.0%)	0.092
Creatinine concentration (µmol/L)	79.1 (66.3–98.4)	98.1 (79.6–123.0)	0.015
Apical ballooning (%)	448 (71.2%)	46 (75.4%)	0.75
Midventricular ballooning (%)	142 (22.6%)	18 (29.5%)	0.29
Basal ballooning (%)	12 (1.9%)	2 (3.3%)	0.80
Focal ballooning (%)	9 (1.4%)	0 (0%)	0.73
Initial EF (%)	43.5 ± 13.5	35.4 ± 13.6	<0.0001
Antihypertensive drugs (%)	533 (84.7%)	45 (73.8%)	0.042
ACE-I or ARB (%)	421 (66,9%)	30 (49.2%)	0.0083
Beta blockers (%)	429 (68.2%)	30 (49.2%)	0.0042
Diuretics (%)	220 (35.0%)	26 (42.6%)	0.29
Aldosterone antagonists (%)	40 (6.4%)	2 (3.3%)	0.50
Statins (%)	301 (48.0%)	20 (32.8%)	0.034
Amiodarone (%)	11 (1.7%)	6 (9.8%)	0.00054
Anticoagulation (%)	172 (27.7'%)	24 (40%)	0.064
Antidiabetic medication (%)	55 (12.2%)	10 (18.9%)	0.25
Thyroid medication (%)	79 (15.3%)	6 (9.8%)	0.35
Levothyroxine (%)	41 (6.5%)	2 (3.3%)	0.47
Perchlorate (%)	6 (1.0%)	3 (4.9%)	0.044
Iodine (%)	2 (0.3%)	0 (0%)	1.00
Antithyroid agents (%)	1 (0.2%)	0 (0%)	1.00
TSH (mIU/L)	1.4 (0.7–2.5)	1.0 (0.4–1.9)	0.022
FT4 (pmol/L)	14.4 (12.6–17.4)	14.9 (12.5–17.0)	0.92
FT3 (pmol/L)	4.2 (2.3–4.8)	3.0 (1.5–4.1)	0.014
SPINA-GT (pmol/s)	3.26 (2.28–6.00)	4.39 (2.50–12.96)	0.075
SPINA-GD (nmol/s)	23.5 (16.2–29.8)	18.5 (10.7–24.8)	0.031
JTI	2.3 (1.6–3.0)	2.0 (1.0–2.6)	0.029
TTSI	66.8 (31.1–119.2)	46.4 815.2–96.1)	0.049
TFQI	0.28 (0.04–0.61)	0.18 (0.00–0.55)	0.088

Table S10: Comparing subjects with and without cardiopulmonary resuscitation (CPR)

	**No CPR (n = 665)**	**CPR (n = 25)**	**p**
Age (years)	70.2 ± 12.5	66.1 ± 15.0	0.16
Female (%)	590 (88.7%)	21 (84%)	0.68
Male (%)	75 (11.3%)	4 (16%)	
BMI (kg/m^2)	24.6 ± 5.0	24.4 ± 5.4	0.67
Hypertension (%)	429 (64.5%)	16 (64.0%)	1.00
Diabetes mellitus (%)	115 (17.3%)	5 (20.0%)	0.93
Atrial fibrillation (%)	104 (15.6%)	8 32.0%)	0.057
Hypercholesterolemia (%)	224 (33.7%)	6 (24%)	0.43
Smoking (%)	142 (21.4%)	8 (32.0%)	0.31
Coronary artery disease (%)	79 (11.9%)	4 (16.0%)	0.76
Malignancy (%)	82 (12.3%)	4 (16.0%)	0.81
Neurological disease (%)	123 (18.5%)	5 (20.0%)	1.00
Psychiatric disease (%)	58 (8.7%)	1 (4.0%)	0.64
Stressful trigger (%)	450 (67.7%)	15 (60.0%)	0.56
Physical trigger (%)	229 (34.4%)	12 (48.0%)	0.24
Emotional trigger (%)	228 (34.3%)	3 (12.0%)	0.036
Creatinine concentration (µmol/L)	79.6 (66.3–101.0)	100.8 (85.3–142.3)	0.0032
Apical ballooning (%)	476 (71.6%)	18 (72.0%)	0.98
Midventricular ballooning (%)	152 (22.9%)	8 (32.0%)	0.41
Basal ballooning (%)	13 (2.0%)	1 (4.0%)	0.41
Focal ballooning (%)	9 (1.4%)	0 (0%)	1.00
Initial EF (%)	43.2 ± 13.5	33.0 ± 15.6	0.001
Antihypertensive drugs (%)	561 (84.4%	17 (68.0%)	0.057
ACE-I or ARB (%)	438 (65.9%)	13 (52.0%)	0.22
Beta blockers (%)	446 (67.1%)	13 (52.0%)	0.18
Diuretics (%)	238 (35.8%)	8 (32.0%)	0.86
Aldosterone antagonists (%)	39 (5.9%)	3 (12.0%)	0.40
Statins (%)	312 (46.9%)	9 (36.0%)	0.38
Amiodarone (%)	13 (2.0%)	4 (16.0%)	0.00015
Anticoagulation (%)	186 (28.4%)	10 (40.0%	0.30
Antidiabetic medication (%)	63 (12.9%)	2 (15.4%)	1.00
Thyroid medication (%)	84 (15.0%)	1 (5.6&)	0.44
Levothyroxine (%)	43 (6.5%)	0 (0%)	0.37
Perchlorate (%)	8 (1.2%)	1 (4.0%)	0.75
Iodine (%)	2 (0.3%)	0 (0%)	1.00
Antithyroid agents (%)	1 (0,2%)	0 (0%)	1.00
TSH (mIU/L)	1.3 (0.7–2.5)	2.0 (0.5–7.7)	0.65
FT4 (pmol/L)	14..5 (12.6–17.4)	15.6 (11.8–17.2)	0.97
FT3 (pmol/L)	4.1 (3.1–4.7)	1.5 (1.5–1.6)	0.10
SPINA-GT (pmol/s)	3.4 (2.3–6.5)	4.3 (2.3–11.5)	0.71
SPINA-GD (nmol/s)	23.1 (15.7–28.9)	11.3(8.0–13.1)	0.11
JTI	2.3 (1.5–3.0)	2.4 (0.9–3.1)	0.78
TTSI	91.9 (27.7–113.7)	100.2 (17.5–137.4)	9.87
TFQI	0.32 (0.03–0.59)	0.38 (0.02–0.66)	0.92

Table S11: Comparing subjects with and without intra-aortic balloon pump (IABP) or extracorporal membrane oxygenation (ECMO) treatment. NA: not available for n = 1.

	**No IABP and ECMO (n = 679)**	**IABP or ECMO (n = 11)**	**P**
Age (years)	70.1 ± 12.5	66.8 ± 16.1	0.51
Female (%)	601 (88.5%)	10 (90.9%)	1.00
Male (%)	78 (11.5%)	1 (9.1%)	
BMI (kg/m^2)	24.6 ± 5.0	25.7 ± 4.7	0.56
Hypertension (%)	436 (64.2%)	9 (81.8%)	0.37
Diabetes mellitus (%)	117 (17.2%)	3 (37.5%)	0.64
Atrial fibrillation (%)			
Hypercholesterolemia (%)	227 (33.4%)	3 (37.5%)	0.91
Smoking (%)	149 (21.9%)	1 (9.1%)	0.51
Coronary artery disease (%)	82 (12.1%)	1 (9.1%)	1.00
Malignancy (%)	86 (12.7%)	0 (0.0%)	0.42
Neurological disease (%)	126 (18.6%)	2 (18.2%)	1.00
Psychiatric disease (%)	59 (8.7%)	0 (0.0%)	0.63
Stressful trigger (%)	460 (67.7%)	5 (45.5%)	0.21
Physical trigger (%)	238 (35.1%)	3 (27.3%)	0.83
Emotional trigger (%)	229 (33.7%)	2 (18.2%)	0.45
Creatinine concentration (µmol/L)	79.6 (66.9–102.4)	103.4 (97.2–119.3)	0.12
Apical ballooning (%)	487 (71.7%)	7 (63.6%)	0.83
Midventricular ballooning (%)	157 (23.1%)	3 (27.3%)	1.00
Basal ballooning (%)	14 (2.1%)	0 (0.0%)	1.00
Focal ballooning (%)	9 (1.3%)	0 (0.0%)	1.00
Initial EF (%)	43.0 ± 13.7	32.2 ± 9.0	0.0039
Antihypertensive drugs (%)	568 (83.7%)	10 (90.9%)	0.81
ACE-I or ARB (%)	445 (65.5%)	6 (54.5%)	0.66
Beta blockers (%)	452 (66.6%)	7 (63.6%)	1.00
Diuretics (%)	240 (35.3%)	6 (54.5%)	0.32
Aldosterone antagonists (%)	42 (6.2%)	0 (0.0%)	0.83
Statins (%)	314 (46.2%)	7 (63.6%)	0.40
Amiodarone (%)	15 (2.2%)	2 (18.2%)	0.016
Anticoagulation (%)	194 (29.0%)	2 (18.2%)	0.65
Antidiabetic medication (%)	64 (13.0%)	1 (11.1%)	1.00
Thyroid medication (%)	85 (14.9%)	0 (0.0%)	0.43
Levothyroxine (%)	43 (6.3%	0 (0.0%)	0.82
Perchlorate (%)	9 (1.3%)	0 (0.0%)	1.00
Iodine (%)	2 (0.3%)	0 (0.0%)	1.00
Antithyroid agents (%)	1 (0.1%)	0 (0.0%)	1.00
TSH (mIU/L)	1.4 (0.7–2.5)	0.6 (0.4–1.5)	0.19
FT4 (pmol/L)	14.5 812.5–17.4)	13.5 (12.3–15.4)	0.67
FT3 (pmol/L)	4.0 (3.1–4.7)	16.9 (NA)	0.091
SPINA-GT (pmol/s)	3.3 (2.3–6.5)	5.7 84.4–8.8)	0.15
SPINA-GD (nmol/s)	22.6 (15.5–28.9)	38.4 (38.4–38.4)	0.12
JTI	2.3 (1.5–3.0)	1.2 (0.5–2.2)	0.072
TTSI	65.2 (28.3–117.8)	24.7 (12.9–55.1)	0.11
TFQI	0.27 (0.03–0.60)	0.00 (–0.16–0.29)	0.12

Table S12: Comparing subjects with and without stroke

	No stroke (n = 661)	Stroke (n = 29)	P
Age (years)	70.2 ± 12.5	68.2 ± 15.3	0.77
Female (%)	587 (88.8%)	24 (82.8%)	0.48
Male (%)	74 (11.2%)	5 (17.2%)	
BMI (kg/m^2)	24.6 ± 5.0	25.0 ± 5.5	0.81
Hypertension (%)	429 64.9%)	16 (55.2%)	0.38
Diabetes mellitus (%)	118 (17.9%)	2 (6.9%)	0.20
Atrial fibrillation (%)	107 (16.2%)	5 (17.2%)	1.00
Hypercholesterolemia (%)	226 (34.2%)	4 (13.8%)	0.038
Smoking (%)	146 (22.1%)	4 (13.8%)	0.41
Coronary artery disease (%)	107 (16.2%)	5 (17.2%)	1.00
Malignancy (%)	82 (12.4%)	4 (13.8%)	1.00
Neurological disease (%)	119 (18.0%)	9 (31.0%)	0.13
Psychiatric disease (%)	56 (8.5%)	3 (10.3%)	0.99
Stressful trigger (%)	441 (66.7%)	24 (82.8%)	0.11
Physical trigger (%)	229 (34.6%)	12 (42.4%)	0.59
Emotional trigger (%)	219 (33.1%)	12 (42.4%)	0.47
Creatinine concentration (µmol/L)	80.2 (67.0–95.1)	82.2 (65.4–114.0)	0.73
Apical ballooning (%)	474 (71.7%)	20 (69.0%)	0.93
Midventricular ballooning (%)	151 (22.8%)	9 (31.0%)	0.42
Basal ballooning (%)	14 (2.1%)	0 (0.0%)	0.91
Focal ballooning (%)	9 (1.4%)	0 (0.0%)	1.00
Initial EF (%)	43.0 ± 13.8	40.0 ± 12.4	0.092
Antihypertensive drugs (%)	553 (83.7%)	25 (86.2%)	0.92
ACE-I or ARB (%)	432 (65.4%)	19 (65.5%)	1.00
Beta blockers (%)	439 (66.4%)	20 (69.0%)	0.93
Diuretics (%)	232 (35.1%)	14 (38.3%)	0.21
Aldosterone antagonists (%)	41 (6.2%)	1 (3.5%)	0.83
Statins (%)	304 (46.0%)	17 (58.6%)	0.25
Amiodarone (%)	14 (2.1%)	3 (10.3%)	0.029
Anticoagulation (%)	179 (27.5%)	17 (58.6%)	0.00065
Antidiabetic medication (%)	64 (13.5%)	1 (3.5%)	0.2
Thyroid medication (%)	81 (14.8%)	4 (13.8%)	1.00
Levothyroxine (%)	41 (6.2%)	2 (6.9%)	1.00
Perchlorate (%)	8 (1.2%)	1 (3.5%)	0.84
Iodine (%)	2 (0.3%)	0 (0.0%)	1.00
Antithyroid agents (%)	1 (0.2%)	0 (0.0%)	1.00
TSH (mIU/L)	1.4 (0.7–2.5)	1.3 (1.0–2.0)	0.87
FT4 (pmol/L)	14.4 (12.5–17.4)	15.3 (13.2–16.5)	0.78
FT3 (pmol/L)	4.1 (3.1–4.7)	1.5 (1.0–4.2)	0.061
SPINA-GT (pmol/s)	3.5 (2.3–6.6)	2.9 (2.3–5.1)	0.62
SPINA-GD (nmol/s)	23.2 (15.8–29.5)	8.4 (7.2–14.8)	0.0071
JTI	2.3 (1.4–3.0)	2.3 (1.9–3.1)	0.50
TTSI	64.5 (23.9–116.7)	62.9 (48.3–106.1)	0.73
TFQI	0.27 (0.03–0.60)	0.28 (0.20–0.89)	0.86

Table S13: Comparing subjects with and without arrhythmia

	No arrhythmia (n = 569)	Arrhythmia (n = 121)	p
Age (years)	70.0 ± 12.5	70.4 ± 13.3	0.52
Female (%)	507 (89.1%)	104 (86.0%)	0.41
Male (%)	62 (10.9%)	17 (14.0%)	
BMI (kg/m^2)	24.5 ± 5.0	24.8 ± 5.2	0.96
Hypertension (%)	361 (63.4%)	84 (69.4%)	0.25
Diabetes mellitus (%)	96 (16.9%)	24 (19.8%)	0.52
Atrial fibrillation (%)	80 (14.1%)	32 (26.4%)	0.0013
Hypercholesterolemia (%)	176 (30.9%)	54 (49.1%)	0.0052
Smoking (%)	133 (23.4%)	17 (14.0%)	0.033
Coronary artery disease (%)	71 (12.5%)	12 (9.9%)	0.53
Malignancy (%)	67 (11.8%)	19 (15.7%)	0.30
Neurological disease (%)	110 (19.3%)	18 (14.9%)	0.31
Psychiatric disease (%)	46 (8.1%)	13 (10.7%)	0.44
Stressful trigger (%)	385 (67.7%)	80 (66.1%)	0.82
Physical trigger (%)	198 (34.8%)	43 (35.5%)	0.96
Emotional trigger (%)	192 (33.7%)	39 (32.2%)	0.83
Creatinine concentration (µmol/L)	77.9 (66.1–100.8)	95.5 (80.0–106.5)	0.0010
Apical ballooning (%)	394 (69.2%)	100 (82.6%)	0.011
Midventricular ballooning (%)	141 (24.8%)	19 (15.7%)	0.042
Basal ballooning (%)	12 (2.1%)	2 (1.6%)	1.00
Focal ballooning (%)	9 (1.6%)	0 (0.0%)	0.34
Initial EF (%)	42.8 ± 13.7	42.7 ± 13.9	0.52
Antihypertensive drugs (%)	474 (83.3%)	104 (86.0%)	0.56
ACE-I or ARB (%)	378 (66.4%)	73 (60.3%)	0.24
Beta blockers (%)	381 (67.0%)	78 (64.5%)	0.27
Diuretics (%)	198 (34.8%)	48 (39.7%)	0.36
Aldosterone antagonists (%)	35 (6.2%)	7 (5.8%)	1.00
Statins (%)	265 (46.6%)	56 (46.3%)	1.00
Amiodarone (%)	6 (1.1%)	11 (9.1%)	<0.0001
Anticoagulation (%)	155 (27.6%)	41 (34.5%)	0.17
Antidiabetic medication (%)	48 (12.1%)	17 (16.0%)	0.36
Thyroid medication (%)	72 (15.5%)	13 (11.6%)	0.38
Levothyroxine (%)	36 (6.3%)	7 (5.8%)	0.99
Perchlorate (%)	5 (0.9%)	4 (3.3%)	0.090
Iodine (%)	2 (0.4%)	0 (0.0%)	1.00
Antithyroid agents (%)	1 (0.2%)	0 (0.0%)	1.00
TSH (mIU/L)	1.3 (0.7–2.5)	1.4 (0.8–2.6)	0.57
FT4 (pmol/L)	14.3 (12.5–17.0)	14.8 (12.8–18.7)	0.092
FT3 (pmol/L)	4.0 (3.1–4.8)	4.2 (2.3–4.8)	0.85
SPINA-GT (pmol/s)	3.2 (2.3–6.6)	3.9 (2.4–6.1)	0.60
SPINA-GD (nmol/s)	22.9 (15.8–28.8)	21.3 (12.1–29.4)	0.57
JTI	2.2 (1.4–2.9)	2.5 (1.5–3.2)	0.18
TTSI	62.4 (26.9–110.9)	70.9 (38.7–144.5)	0.26
TFQI	0.26 (0.03–0.59)	0.29 (0.07–0.60)	0.20

Table S14: Univariable and hierarchical models from Cox regression for the composite endpoint of in-hospital complications (arrhythmias, thromboembolic events or stroke, pulmonary congestion, cardiogenic shock using catecholamine or assist device). Hierarchical analysis is based on sex and age as frailty clusters.

	**Univariable HR (95% CI)**	**p**	**Hierarchical HR (95% CI**	**p**
TSH (mIU/L)	1.00 (0.91–1.10)	1.00	1.01 (0.91–1.11)	0.88
FT4 (pmol/l)	1.00 (0.98–1.03)	0.72	1.01 (0.98–1.03)	0.54
FT3 (pmol/L)	1.05 (0.95–1.16)	0.30	0.07 (0.97–1.19)	0.15
Age (years)	1.00 (0.99–1.00)	0.65		
Female sex	0.71 (0.49–1.03)	0.069		
Hypertension	0.88 (0.66–1.17)	0.37	0.87 (0.65–1.16)	0.33
Diabetes mellitus	0.94 (0.66–1.31)	0.69	0.93 (0.66–1.31)	0.66
Hypercholesterolemia	1.28 (0.97–1.70)	0.082	1.28 (0.96–1.70)	0.091
Smoking	0.81 (0.57–1.15)	0.26	0.79 (0.55–1.12)	0.19
Atrial fibrillation	1.05 (0.76–1.45)	0.76	1.04 (0.75–1.43)	0.82
Malignancy	0.90 (0.61–1.32)	0.59	0.88 (0.60–1.30)	0.53
Neurological disease	1.07 (0.77–1.47)	0.70	1.08 (0.78–1.49)	0.65
Psychiatric disease	1.01 (0.63–1.63)	0.96	1.03 (0.64–1.67)	0.89
Stressful trigger	1.14 (0.85–1.53)	0.37	1.14 (0.85–1.53)	0.37
Physical trigger	1.02 (0.77–1.35)	0.92	1.00 (0.76–1.33)	0.98
Emotional trigger	1.23 (0.91–1.66)	0.18	1.24 (0.92–1.68)	0.16
Creatinine (µmol/L)	1.00 (1.00–1.01)	0.023	1.00 (1.00–1.01)	0.026
Apical ballooning	1.01 (0.73–1.39)	0.95	1.00 (0.72–1.38)	0.99
Midvent. ballooning	1.04 (0.74–1.45)	0.83	1.04 (0.74–1.45)	0.82
Basal ballooning	1.19 (0.44–3.21)	0.73	1.16 (0.43–3.14)	0.77
Focal ballooning	0.00 (0.00–∞)	1.00	0-00 (0.00–∞)	1.00
Initial EF (%)	1.00 (0.99–1.01)	0.64	1.00 (0.99–1.01)	0.69

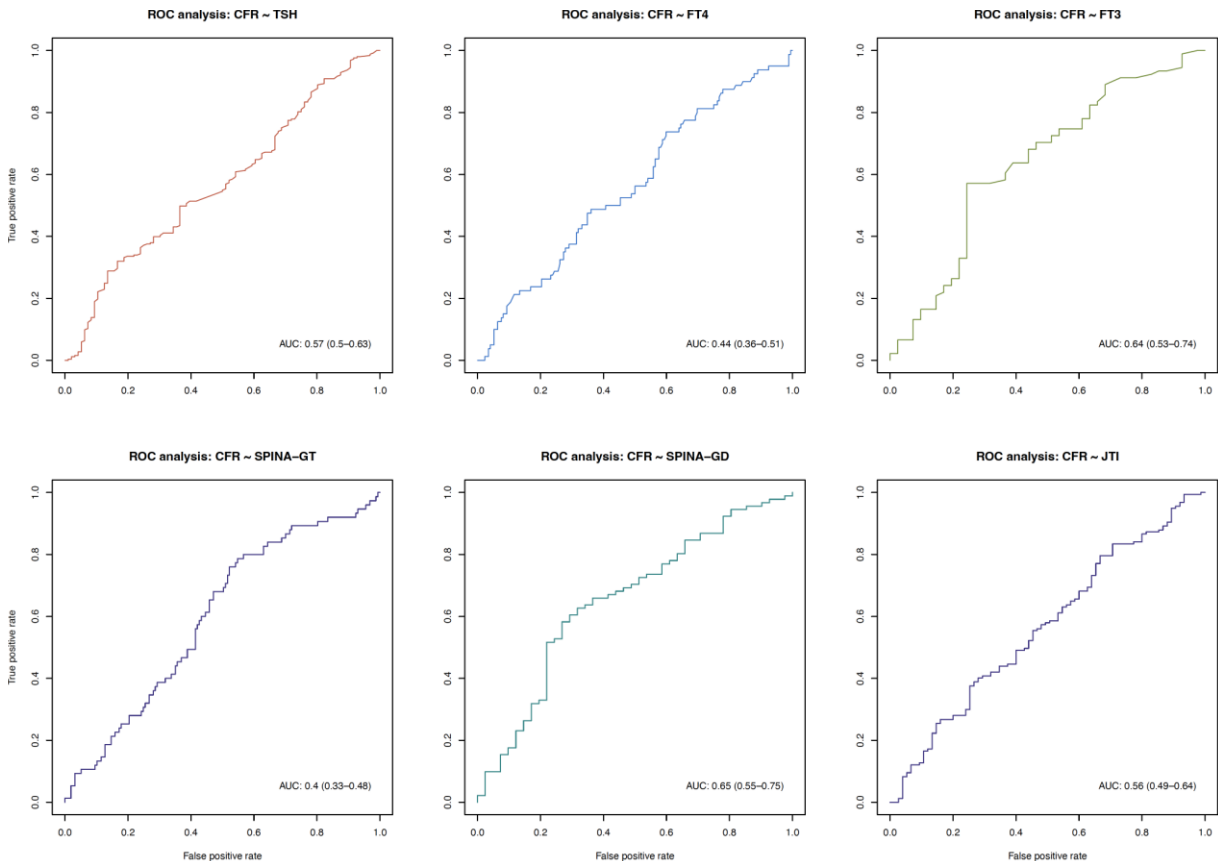

Figure S2: Receiver operating characteristics (ROC) curves for measured and calculated biomarkers of thyroid homeostasis.

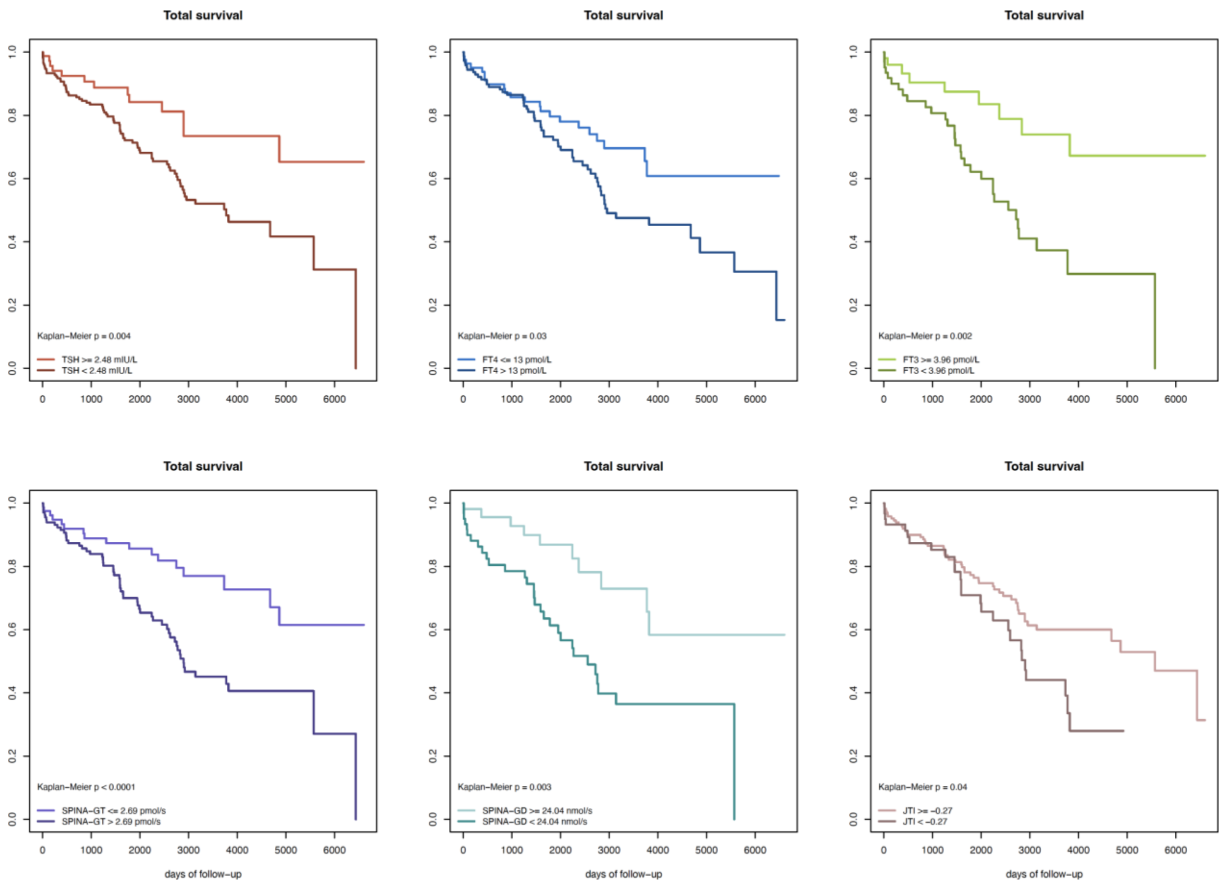

Figure S3: Kaplan-Meier survival curves based on cutoff values derived from Youden's *J*.

Supplementary References

1. Dietrich JW, Landgrafe-Mende G, Wiora E, et al. Calculated Parameters of Thyroid Homeostasis: Emerging Tools for Differential Diagnosis and Clinical Research. *Front Endocrinol (Lausanne)* 2016; **7**: 57.
2. Jostel A, Ryder WD, Shalet SM. The use of thyroid function tests in the diagnosis of hypopituitarism: definition and evaluation of the TSH Index. *Clin Endocrinol (Oxf)* 2009; **71**(4): 529-34.
3. Pohlenz J, Weiss RE, Macchia PE, et al. Five new families with resistance to thyroid hormone not caused by mutations in the thyroid hormone receptor beta gene. *J Clin Endocrinol Metab* 1999; **84**(11): 3919-28.
4. Yagi H, Pohlenz J, Hayashi Y, Sakurai A, Refetoff S. Resistance to thyroid hormone caused by two mutant thyroid hormone receptors beta, R243Q and R243W, with marked impairment of function that cannot be explained by altered in vitro 3,5,3'-triiodothyroinine binding affinity. *J Clin Endocrinol Metab* 1997; **82**(5): 1608-14.
5. Laclaustra M, Moreno-Franco B, Lou-Bonafonte JM, et al. Impaired Sensitivity to Thyroid Hormones Is Associated With Diabetes and Metabolic Syndrome. *Diabetes Care* 2019; **42**(2): 303-10.
6. Kohl M. MKpower-package: Power Analysis and Sample Size Calculation. 0.7 ed; 2023.
